# Supplementary material for: Enrichment of FGF8-expressing cells from neurally induced human pluripotent stem cell cultures
Source: Stem Cell Reports. 2023 Nov 2;18(11):2240–53. doi: 10.1016/j.stemcr.2023.10.007 (PMC10679777; doi:10.1016/j.stemcr.2023.10.007)
Supplement: Document S1. Supplemental experimental procedures, Figures S1–S4, and Tables S1–S4 [file mmc1.pdf]

Stem Cell Reports, Volume *18*

Supplemental Information

Enrichment of FGF8-expressing cells from neurally induced human pluripotent stem cell cultures

Nils Offen, Alina Filatova, and Ulrike A. Nuber

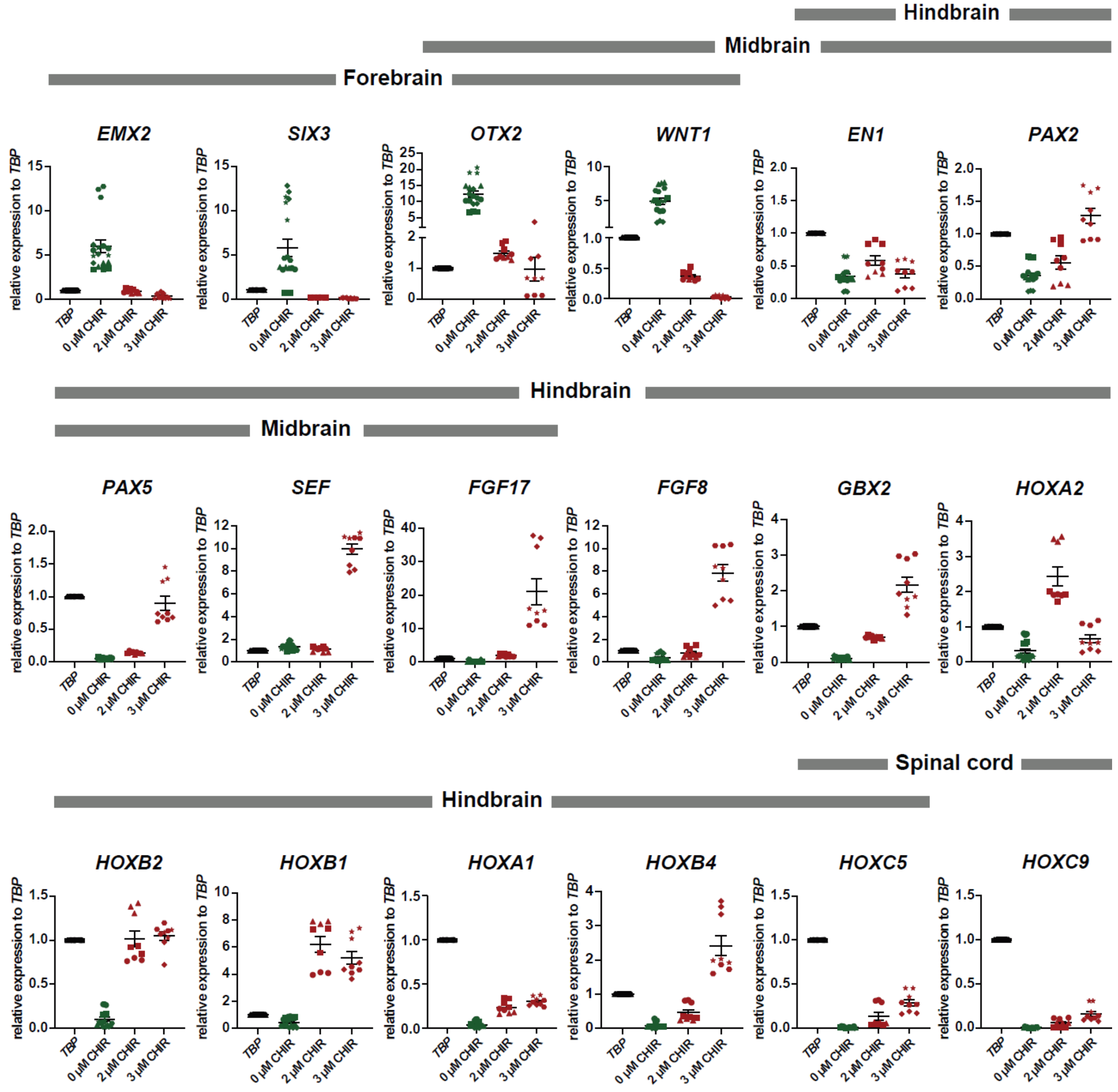

Figure S1. Transcript levels of genes along the anterior-posterior neural tissue axis in neurally induced and attached hESC-H9 EBs treated with 2 or 3 µM CHIR99021 in comparison to untreated samples (0 µM CHIR99021).

Quantitative RT-PCR data from three independent experiments (three independent experiments with 0 and 2 µM CHIR99021 are displayed as triangles, circles, and boxes; three independent experiments with 0 and 3 µM CHIR99021 are displayed as diamonds, stars, and hexagons). For each independent biological replicate, three technical replicates (RT-qPCR experiments) were performed. Horizontal bars display the mean values, vertical bars represent standard errors. Transcript levels of the *TBP* reference gene are set to 1. Note that these genes are not exclusively expressed in indicated brain regions.

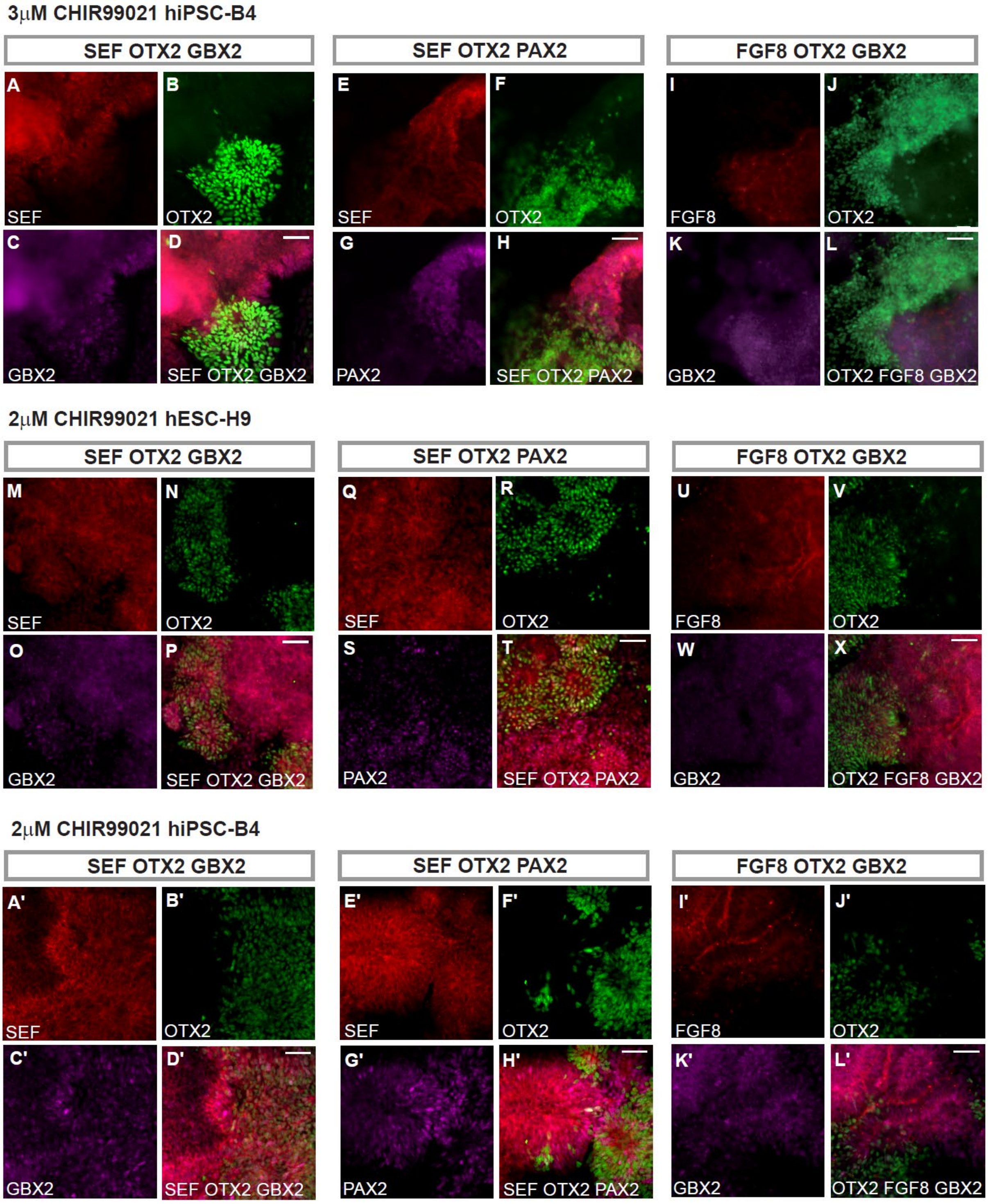

Figure S2. **Distribution of SEF, OTX2, GBX2, PAX2, and FGF8 proteins in neurally induced, caudalized attached EBs studied by co-immunofluorescence stainings**.

Overlay images are shown in the lower right corner of each panel.

(A-L) 3 μM CHIR99021 treated hiPSC-B4, (M-X), 2 μM CHIR99021 treated hESC-H9, (A'-L') 2 μM CHIR99021 treated hiPSC-B4.

In 3 μM CHIR99021 treated hiPSC-B4 cultures, SEF-positive regions are largely overlapping with GBX2- and PAX2-positive areas (A, C, E, G) and are largely distinct from OTX2-positive

areas (D, H). Few OTX2-positive cells are found in the GBX2-, SEF-, and PAX2-positive regions (D, H, L). FGF8-positive regions are present within the GBX2-positive areas (I, K).

In 2 µM CHIR99021 treated hESC-H9 and hiPSC-B4 cultures, SEF-positive regions overlap with GBX2-positive (M, O, A', C') and PAX2-positive areas (Q, S, E', G'). A more extensive overlap of OTX2-positive regions with GBX2-positive/SEF-positive regions is present (M-P, A'-D'). FGF8 immunosignals are detected in GBX2-positive, but also in OTX2-positive areas (U-W, I'-K'). These representative images are derived from three independent experiments per cell line and per CHIR99021 treatment condition with three technical replicates each. Scale bars: 50 µm.

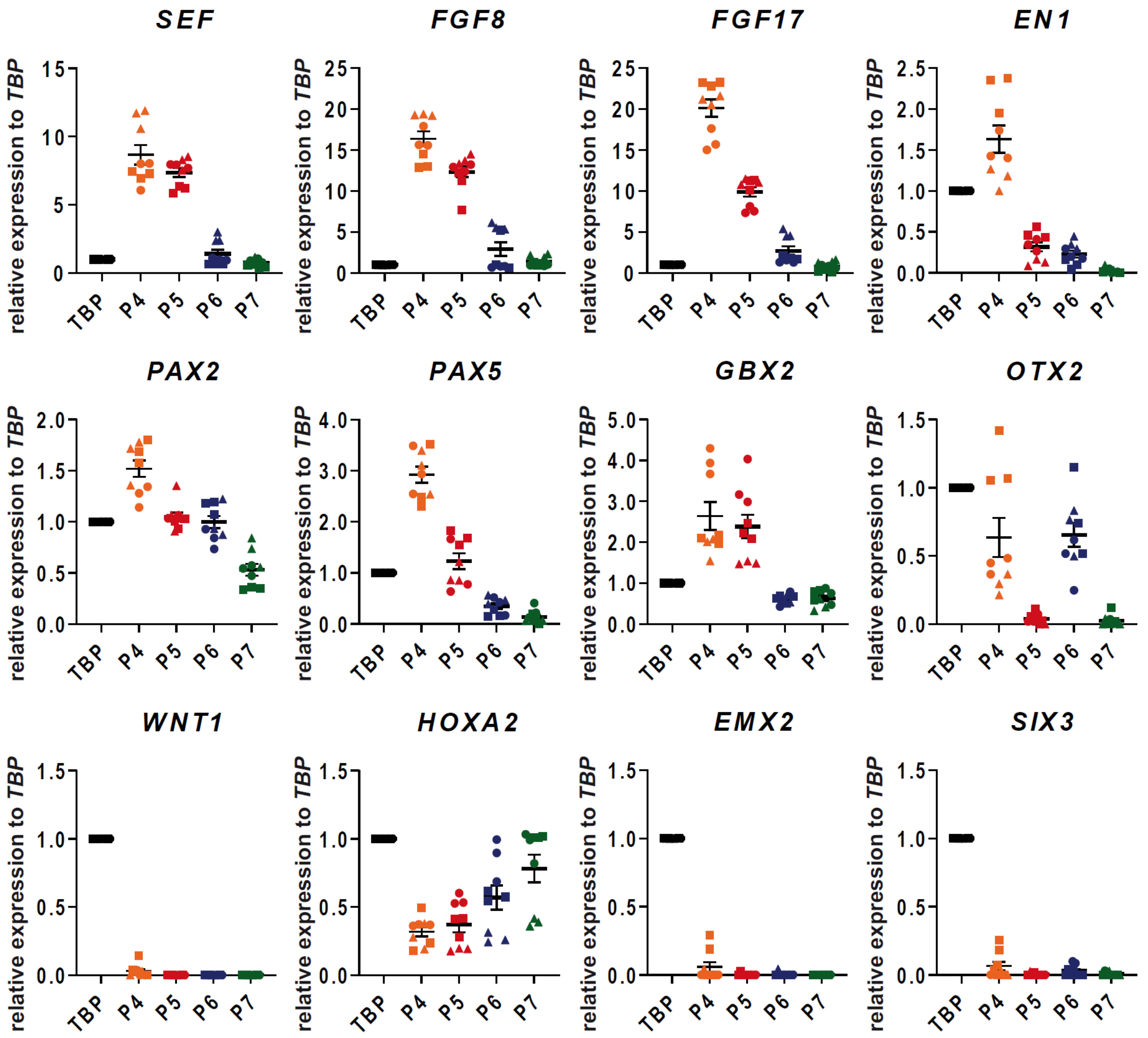

Figure S3. The SEF^{high}/18R5^{low} population isolated from neurally induced, 3 µM CHIR99021 caudalized and attached hiPSC-B4 EBs is enriched for midbrain-hindbrain organizer gene transcripts.

Quantitative RT-PCR analyses of the flow cytometry-sorted cell populations SEF^{high}/18R5^{low} (P4), SEF^{high}/18R5^{high} (P5), SEF^{low}/18R5^{low} (P6), and SEF^{low}/18R5^{high} (P7). Transcript levels of

the reference gene *TBP* are set to 1. Data from three independent experiments (independent biological replicates) are displayed as filled triangles, circles and boxes. For each independent biological replicate, three technical replicates (RT-qPCR experiments) were performed. Horizontal bars display the mean values, vertical bars represent standard errors.

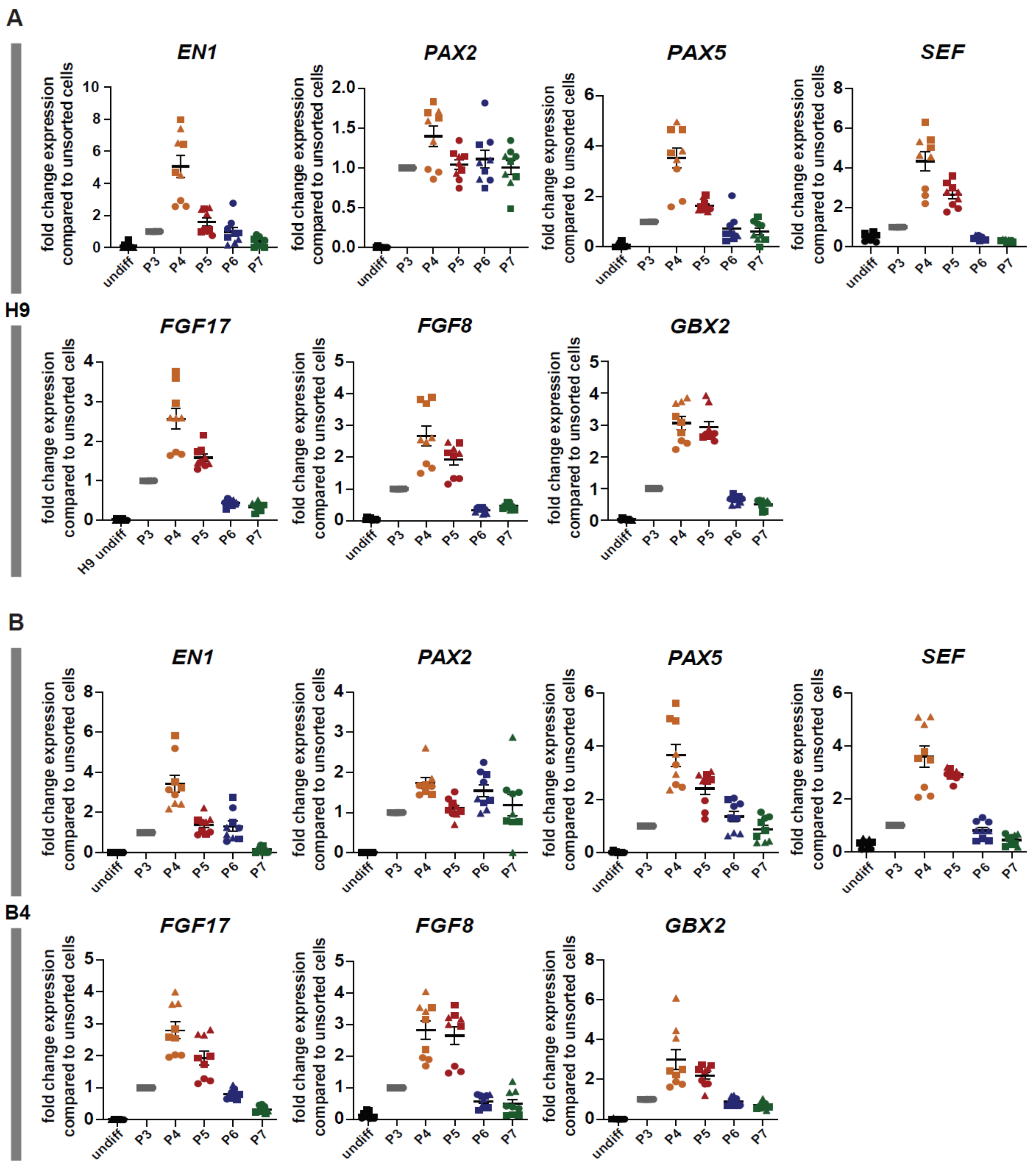

Figure S4. Transcript levels of genes in populations P4, P5, P6, and P7 from 3 µM CHIR99021 treated cultures displayed as fold changes in comparison to the total unsorted population P3 from these cultures.

Quantitative RT-PCR analyses of the flow cytometry-sorted cell populations SEF^{high}/18R5^{low} (P4), SEF^{high}/18R5^{high} (P5), SEF^{low}/18R5^{low} (P6), and SEF^{low}/18R5^{high} (P7), unsorted cell

population (P3), and undifferentiated hPSCs. Data from three independent experiments (independent biological replicates) of hESC-H9 (A) and hiPSC-B4 (B) are displayed as filled triangles, circles, and boxes. For each independent biological replicate, three technical replicates (RT-qPCR experiments) were performed. Horizontal bars display the mean values, vertical bars represent standard errors.

Supplemental experimental procedures

Maintenance of human pluripotent stem cell (hPSC) lines

Lines were weekly passaged and tested for mycoplasma contamination. For passaging, colonies were treated for 5 min with 1.5 mg/ml Collagenase IV in DMEM/F12 at room temperature and detached using a cell scraper. For detailed information on media and supplements, see Table S1.

Preparation of mouse embryonic fibroblast (MEF) feeder cells

MEFs at passage 3 were treated with 10 µg/ml Mitomycin C for 2 h, washed twice with DMEM medium and detached for 5 min with Trypsin-EDTA at 37°C. 4x 10^6 cells per vial were frozen in a 1:1 mixture of MEF medium and FBS supplemented with 10% DMSO. For feeder layers, cells were thawed and replated onto 60 mm cell culture dishes coated with 0.2% gelatin in DPBS for 30 min at 37°C in MEF medium at a seeding density of 30,000-35,000 cells/cm^2. For detailed information on media and supplements, see Table S1.

Neural differentiation of hPSC lines - coating of plates and cover slips

Coating of the bottom of 6 well plates or of 12 mm cover slips (Epredia, CB00120RA120MNZ0) in 24 well plates was performed as follows. 15 µg/ml Poly-ornithine in DPBS (1 ml in case of 6-well plates or 0.5 ml in case of 24-well plates) was added to each well and incubated at 37°C for at least 12 h). After the removal of the poly-ornithine solution, each well was washed twice with 1 (6-well plates) or 0.5 ml (24-well plates) DPBS, respectively. DPBS was removed and DPBS supplemented with 50 µg/ml laminin and 50 µg/ml fibronectin was added to each well (1 ml for 6-well and 0.5 ml for 24-well plates). Plates were incubated at 37°C for 24-48 h, washed twice with either 1 ml (6-well plates) or 0.5 ml (24-well plates) DPBS, and used within a week. For detailed information on media and supplements, see Table S1.

Total RNA extraction

Cells were dissociated into single cells by a 6 min Accutase (Table S1) incubation at 37°C. Cells were rinsed once with DPBS and afterwards either frozen in liquid nitrogen and stored at -80 °C or processed immediately. Total RNA was extracted using the Quick-RNA™ MiniPrep kit (Table S4) according to the manufacturer’s instructions.

RT-qPCR analysis

RT-qPCR data were analyzed using the StepOne software v2.3 (Thermo Fisher Scientific) and Microsoft Excel (Microsoft). RT-qPCR data shown in Figures 2, S1 and S3 were normalized to the reference gene *RPL13A* and presented as $2^{-\Delta CT}{}_{Marker}$ relative to $2^{-\Delta CT}{}_{TBP}$ ± SEM using GraphPad Prism9 (GraphPad Software Inc.). RT-qPCR data shown in Figure S4 were analyzed using the ΔΔCT method. Samples were normalized to the reference gene *RPL13A* and the population P3 (unsorted cells) served as reference population. Data was presented as mean fold change ± SEM compared to the P3 population using GraphPad Prism9.

***In situ* hybridization of paraffin tissue sections**

The following plasmids were used to generate RNA *in situ* probes: *En1* (from Susanne Dietrich), *Fgf8* (from Astrid Vogel-Höpker), *Gbx2* (from Jose Luis Ferran), *Wnt1* (from Clemens Kiecker) and *Sef*. To generate the *Sef* encoding plasmid, a 530 bp cDNA fragment corresponding to nucleotides 1016-1545 of Genbank: XM_040646074.1 was PCR-amplified using a forward (5'-TCTAGAATTCTCCCTGTG GAAAGACTTCGC-3') and reverse primer (5'-TTTCTAGAACACCTCTGAATCCTGC ACAC-3') and cloned into pBluescriptII SK+ via *BamHI* and *XbaI* sites. All plasmids were sequenced to confirm respective cDNA inserts.

To generate Dig-labeled RNA probes, a DNA fragment with a corresponding cDNA sequence of interest and the T3, SP6 or T7 promoter sequence, was amplified by PCR. PCR amplification was performed in 50 µl reactions containing 200 nM of forward (5'-CCTCTTCGCTATTACGCCAG-3') and reverse primer (5'-GGCTCGTATGTTGTGTGGAA-3'), 200 µM dNTPs and 0.2 U/µl Q5 High Fidelity DNA polymerase in an GeneAmp 9600 PCR cycler (Applied Biosystems) under the following conditions. 2 min 98°C, followed by 35 cycles 10 sec 98°C, 20 sec 56°C and 1 min 30 sec 72°C and a final elongation step for 2 min at 72°C. PCR products were purified using the DNA Clean & Concentrator-5 kit according to the manufacturer's instructions. 1 µg of DNA was used for *in vitro* transcription with the following RNA polymerases: *En1* and *Sef,* T7; *Fgf8* and *Wnt1*, T3; *Gbx2*, Sp6.

Measurement of optic lobe length

Lateral photos of isolated embryonic chicken brains transplanted with either collagen-$SEF^{high}/18R5^{low}$ cell particles or collagen-only were taken at day 6 post transplantation

using a Nikon SMZ1500 stereoscopic zoom microscope. Fiji (Schindelin et al., 2012) was used to measure the rostro-caudal length of the whole brain and of the optic lobe. Data were presented as ratios of optic lobe length to whole brain length using Graphpad Prism.

Table S1: Media and supplements used for cell culture and FACS.

Medium/Supplement	Distributor	Cat. No.
Accutase	Sigma-Aldrich	A6964
Albumin Fraktion V (BSA)	Carl Roth	CP84.2
B27 supplement without vitamin A (50x)	Thermo Fisher Scientific	12587-010
ß-mercaptoethanol	Thermo Fisher Scientific	31350-010
Collagen, Type I solution from rat tail (3 mg/ml)	Sigma-Aldrich	C3867
Collagenase IV	Thermo Fisher Scientific	17104-019
CHIR99021	Sigma-Aldrich	SML1046
DMEM high Glucose	Sigma-Aldrich	D5671
DMEM/F12	Sigma-Aldrich	51445C
DPBS	Sigma-Aldrich	D8537
DMSO	Sigma-Aldrich	D4540
UltraPure™0.5 M EDTA, pH 8.0	Thermo Fisher Scientific	15575020
BD FACSFlow™ Sheath Fluid	BD Biosciences	342003
FBS	Sigma-Aldrich	F7524
Recombinant human FGF basic (154 a.a.)	Peprotech	100-18B
Fibronectin	Thermo Fisher Scientific	33010-018
Gelatin	Sigma-Aldrich	G1890
Gentamycin	Sigma-Aldrich	G1397
1M HEPES solution	Thermo Fisher Scientific	15630-080
KnockOut Serum Replacement	Thermo Fisher Scientific	10828-010
Laminin	Sigma-Aldrich	L2020
LDN193189 hydrochloride	Sigma-Aldrich	SML0559
L-glutamine	Sigma-Aldrich	G7513
MEM non-essential amino acids	Sigma-Aldrich	M7145
Mitomycin C	Roche	10107409001
N2 supplement (100x)	Thermo Fisher Scientific	17502-048
Neurobasal medium	Thermo Fisher Scientific	21103-049
Penicillin-Streptomycin	Sigma-Aldrich	P0781

Poly-L-ornithine hydrobromide	Sigma-Aldrich	P3655
RevitaCell™ supplement (100x; Rho-associated kinase inhibitor)	Thermo Fisher Scientific	A2644501
StemMACS™ SB431542	Miltenyi Biotec	130-106-275
SYTOX™ Red dead cell stain	Thermo Fisher Scientific	S34859
0.5% Trypsin/ 0.2% EDTA	Sigma-Aldrich	T3924

Table S2: Primary and secondary antibodies.

**Antibody**	**Distributor**	**Cat. No.**	**Stock concen-tration**	**Dilution for immuno-stainings**
**Primary antibodies**				
sheep anti-Digoxigenin-AP, RRID:AB_2313640	Roche	11 093 274 910	0.75 U/µl	1:1500
mouse anti-FGF8, RRID:AB_2927654	Creative Biolabs	NEUT-823CQ	0.5 mg/ml	1:500
human anti-FZD (Vantictumab), RRID:AB_2911149	Thermo Fisher Scientific	MA5-42006	1.1 mg/mL	1:10000
rabbit anti-GBX2, RRID:AB_2685900	Sigma-Aldrich	HPA067809	900 µg/ml	1:500
mouse anti-MEIS2, RRID:AB_2618842	DSHB	PCRP-MEIS2-1A11	21 µg/ml	1:10
goat anti-OTX2, RRID:AB_2157172	R&D Systems	AF1979	200 µg/ml	1:500
mouse anti-PAX2, RRID:AB_2722284	DSHB	PCRP-PAX2-1A7	12 µg/ml	1:10
rabbit anti-PAX2, RRID:AB_2734656	Biolegend	901002	1 mg/ml	1:500
rabbit anti-PAX6, RRID:AB_2565003	Biolegend	901301	2 mg/ml	1:500
mouse anti-IL-17RD/SEF, RRID:AB_2125688	R&D Systems	MAB2275	250 µg/ml	1:200
rabbit anti-TCF4/TCF7L2, RRID:AB_2199816	Cell Signaling Technology	2569	-	1:200
Rat anti-mouse feeder cells antibody, RRID:AB_2752027	Miltenyi Biotec	130-120-166	150 µg/ml	only used for FACS
Mouse anti-human nuclei antibody, RRID:AB_94090	EMD Millipore	MAB1281	-	1:200

**Secondary antibodies**				
anti-goat-AF488, RRID:AB_2336933	Dianova	705-545-147	1 mg/ml	1:500
anti-goat-AF647, RRID:AB_2535864	Thermo Fisher Scientific	A-21447	1 mg/ml	1:500
anti-human-AF488, RRID:AB_2340565	Dianova	709-545-098	1 mg/ml	FACS only
anti-mouse-Cy3, RRID:AB_2340813	Dianova	715-165-150	1 mg/ml	1:300-1:500
anti-rabbit-Cy5, RRID:AB_2340607	Dianova	711-175-152	1 mg/ml	1:500-1:1000
anti-goat-Cy3, RRID:AB_2307351	Dianova	705-165-147	1 mg/ml	1:300
anti-mouse DyLight 488, RRID:AB_2687442	Dianova	715-485-150	1.5 mg/ml	1:300
anti-rabbit-AF488, RRID:AB_2313584	Dianova	711-545-152	1 mg/ml	1:300
anti-mouse-RPE, RRID:AB_2338622	Dianova	115-115-207	1 mg/ml	only used for FACS
**Flow cytometry isotype control**				
mouse IgG2B isotype control, RRID:AB_357346	R&D Systems	MAB004	1 mg/ml	only used for FACS

Table S3: RT-qPCR primers.

Gene	Primer	Sequence (5' → 3')
EMX2	EMX2-fw	GGATCCGTCCACCTTCTACC
	EMX2-rev	CGTGTTCCAGCCTTAGAAGC
EN1	EN1- fw	CTGCACACGTTATTCGGATCG
	EN1-rev	CGCTTGTCCTCCTTCTCGTTC
FGF8	FGF8-fw	CAACTCTACAGCCGCACCAG
	FGF8-rev	TGCTTCCAAAGGTGTCCGTC
FGF17	FGF17-fw	GCAGATCCGCGAGTACCAAC
	FGF17-rev	GTCCGTCTCCACTATGAGCTTG
GBX2	GBX2-fw	AGGACGGCAAAGGCTTCCTG
	GBX2-rev	GACTCGTCTTTCCCTTGCC
HOXA1	HOXA1-fw	TCGCCTCAATACATTCACCACT
	HOXA1-rev	TTGACCCAGGTAGCCGTACT
HOXA2	HOXA2-fw	CTTGCCTCAGCCACAAAGAATC
	HOXA2-rev	GCTGTGTGTTGGTGTAAGCAG
HOXB1	HOXB1-fw	CAGAACCTAACACCCCCACG
	HOXB1-rev	AGTTCTGTCAGCTGCCTTGT
HOXB2	HOXB2-fw	AATCCGCCACGTCTCCTTC
	HOXB2-rev	CAGCTGCGTGTTGGTGTAAG
HOXB4	HOXB4-fw	CTACCCCTGGATGCGCAAAG
	HOXB4-rev	GTCAGGTAGCGGTTGTAGTGA
HOXC5	HOXC5-fw	TACCCGTGGATGACCAAACTG
	HOXC5-rev	GAGACACAAGTTGTTGGCGA
HOXC9	HOXC9-fw	CCGGCAGCAAGCACAAAGA
	HOXC9-rev	TACCGACGGTCCCTGGTTAAA

OTX2	OTX2-fw	CGGTACCCAGACATCTTCATG
	OTX2-rev	CGGCACTTAGCTCTTCGATTC
PAX2	PAX2-fw	GACTATGTTCGCCTGGGAGATTC
	PAX2-rev	AAGGCTGCTGAACTTTGGTCCG
PAX5	PAX5-fw	GTAGTCCGCCAGAGGATAGTG
	PAX5-rev	TCCAATTACCCCAGGCTTGATG
RPL13A	RPL13A-fw	GTATGCTGCCCCACAAAACC
	RPL13A-rev	TTCAGACGCACGACCTTGAG
SEF	SEF-fw	CGACGCTCTTCACTGTGATGTG
	SEF-rev	GCTGCAGTGTATGTGGAAGAC
SIX3	SIX3-fw	AGAACAGGCTCCAGCACCAG
	SIX3-rev	CTGGAGGTTACCGAGAGGATG
TBP	TBP-fw	CTTGTGCTCACCCACCAAC
	TBP-rev	CTGCTCTGACTTTAGCACCTG
WNT1	WNT1-fw	CCGATGGTGGGGTATTGTGA
	WNT1-rev	TCCCCGGATTTTGGCGTATC

Table S4: Enzymes and commercial kits used for *in situ* hybridization probe generation and total RNA extraction.

**Enzyme**	**Distributor**	**Cat. No.**
Q5 High Fidelity DNA polymerase	NEB	M0491S
SP6 Polymerase	New England Biolabs (NEB)	M0207S
T3 Polymerase	New England Biolabs (NEB)	M0378S
T7 Polymerase	New England Biolabs (NEB)	M0251S
DNA Clean & Concentrator-5 kit	Zymo Research	D4004
Quick-RNA™ MiniPrep kit	Zymo Research	R1054
RNeasy Mini Kit	Qiagen	74104

Reference

Schindelin, J., Arganda-Carreras, I., Frise, E., Kaynig, V., Longair, M., Pietzsch, T., Preibisch, S., Rueden, C., Saalfeld, S., Schmid, B., *et al.* (2012). Fiji: an open-source platform for biological-image analysis. Nat Methods *9*, 676-682.
