# Supplementary material for: Enrichment of FGF8-expressing cells from neurally induced human pluripotent stem cell cultures
Source: Stem Cell Reports. 2023 Nov 2;18(11):2240–53. doi: 10.1016/j.stemcr.2023.10.007 (PMC10679777; doi:10.1016/j.stemcr.2023.10.007)
Supplement: Document S2. Article plus supplemental information [file mmc2.pdf]

Stem Cell Reports
Article
ISSCR
OPEN ACCESS

Enrichment of FGF8-expressing cells from neurally induced human pluripotent stem cell cultures

Nils Offen,[1] Alina Filatova,[1] and Ulrike A. Nuber[1,*]
[1]Stem Cell and Developmental Biology, Technical University of Darmstadt, 64287 Darmstadt, Germany
*Correspondence: nuber@bio.tu-darmstadt.de
https://doi.org/10.1016/j.stemcr.2023.10.007

SUMMARY

In early vertebrate development, organizer regions—groups of cells that signal to and thereby influence neighboring cells by secreted morphogens—play pivotal roles in the establishment and maintenance of cell identities within defined tissue territories. The midbrain-hindbrain organizer drives regionalization of neural tissue into midbrain and hindbrain territories with fibroblast growth factor 8 (FGF8) acting as a key morphogen. This organizer has been extensively studied in chicken, mouse, and zebrafish. Here, we demonstrate the enrichment of *FGF8*-expressing cells from human pluripotent stem cells (hPSCs), cultured as attached embryoid bodies using antibodies that recognize "Similar Expression to *Fgf*" (SEF) and Frizzled proteins. The arrangement of cells in embryoid body subsets of these cultures and the gene expression profile of the *FGF8*-expressing population show certain similarities to the midbrain-hindbrain organizer in animal models. In the embryonic chick brain, the enriched cell population induces formation of midbrain structures, consistent with FGF8-organizing capability.

INTRODUCTION

Organizers—groups of cells that induce neighboring cells to acquire specific fates and to form organized structures—steer the development of animal tissues and organs. This key principle of animal development was first discovered at the beginning of the 20th century in Hydra and in newt embryos by grafting region-specific donor tissue to a different site in recipients and monitoring grafts for organizer activity that induced recipient cells to develop into new tissue structures (Browne, 1909; Spemann and Mangold, 1924). The organizer acts on neighboring cells through tissue-specific morphogens such as proteins and steroids in a concentration-dependent manner (Anderson and Stern, 2016; Martinez Arias and Steventon, 2018). Morphogen effects are intimately linked with the developmental state of cells, i.e., their competence to respond to specific signals. Four regions are among the unambiguously and best characterized organizers to date: the primary or Mangold-Spemann organizer (known as Hensen's node in amniotes), the notochord, the limb bud polarizing activity zone, and the midbrain-hindbrain boundary (MHB) (Anderson and Stern, 2016). The latter three are considered secondary organizers because they emerge and act at later developmental stages after gastrulation, during which the primary organizer is active.

The midbrain-hindbrain organizer is located at the boundary between the developing midbrain (mesencephalon) and the anterior part (rhombomere 1) of the hindbrain (metencephalon) (Gibbs et al., 2017; Harada et al., 2016; Leto et al., 2016; Martinez et al., 2013; Raible and Brand, 2004; Wurst and Bally-Cuif, 2001; Zervas et al., 2005). Because of the narrowing (isthmus) of the neural tube at this site, it is also called the isthmic organizer. This organizer was initially identified through transplantation experiments with quail and chicken embryos. In seminal work, it was found to induce the prospective diencephalon (caudal forebrain) to undergo a switch toward a midbrain fate (Marin and Puelles, 1994; Martinez et al., 1991; Nakamura et al., 1986), whereas transplantation into the hindbrain caudal to rhombomere 1 resulted in a fate switch toward a cerebellum fate (Martinez et al., 1995). Subsequent gain and loss-of-function studies established that fibroblast growth factor 8 (FGF8) is a key secreted morphogen mediating midbrain-hindbrain organizer functions in chicken, mouse, and zebrafish (Crossley et al., 1996; Harada et al., 2016; Lee et al., 1997; Liu et al., 1999; Martinez et al., 1999; Partanen, 2007; Sato et al., 2001). Early patterning at the MHB occurs through two main transcription factors: OTX2 in the anterior part (presumptive forebrain and midbrain) and GBX2 in the posterior part (presumptive hindbrain). In later development, genes encoding the transcription factors PAX2, PAX5, and EN1 and the secreted factors WNT1 and FGF8 are activated at the boundary between the *Otx2-Gbx2* domains in mouse and chicken (Rhinn and Brand, 2001). The *Otx2*, *Gbx2*, *Wnt1*, and *Fgf8* expression domains are initially overlapping and, from Hamburger Hamilton (HH)11–12 in chicken and E10 in mouse, become sharply separated territories with *Wnt1* expressed in the caudal-most portion of the *Otx2* domain, immediately adjacent to *Fgf8* expressed in the rostral-most *Gbx2* domain (Leto et al., 2016) (Figures 1A–1J). *Pax2/5* and *En1/2* expression fields span the *Otx2-Gbx2* domain border (Leto et al., 2016; Zervas et al., 2005) (Figures 1A–1J). As a key morphogen, FGF8 establishes and maintains the *Otx2-Gbx2* boundary through

This is an open access article under the CC BY license (http://creativecommons.org/licenses/by/4.0/).

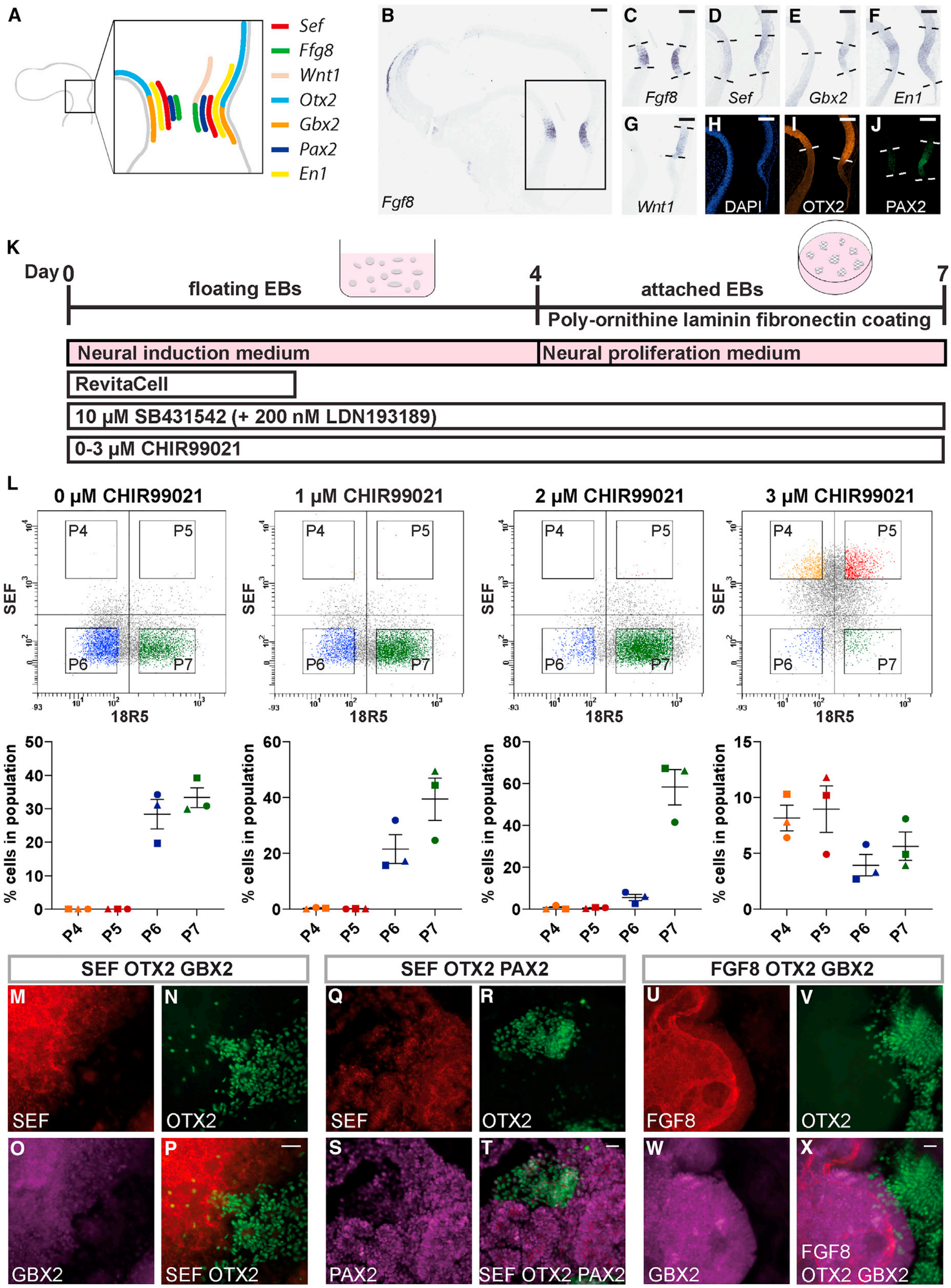

(legend on next page)

a complex gene regulatory network that specifies cell identities and positions by defining gene expression patterns (Partanen, 2007; Raible and Brand, 2004; Sunmonu et al., 2011; Wurst and Bally-Cuif, 2001; Zervas et al., 2005).

Here, using human pluripotent stem cells, we generate *FGF8*-expressing cells that show certain similarities to midbrain-hindbrain organizer tissue and exert midbrain-organizing effects in the chick embryo.

RESULTS

A *SEF*-expressing human stem cell-derived population

Our data demonstrate that *FGF8*-expressing cells that show certain similarities to midbrain-hindbrain organizer tissue can be isolated using flow cytometry from neurally induced and caudalized human embryonic stem cell (hESC) and human induced pluripotent stem cell (hiPSC) cultures, hereafter referred to as human pluripotent stem cells (hPSCs), using a combination of two antibodies recognizing "Similar Expression to *Fgf*" (SEF) and certain Frizzled (FZD) cell surface proteins. *SEF* (synonym: *IL17RD*) was first identified in zebrafish and is induced by different FGFs, including FGF8 (Furthauer et al., 2002; Ron et al., 2008; Tsang et al., 2002). Although SEF inhibits FGF signaling and downstream effects during development, the combined expression of *Fgf8* and *Sef* leads cells to produce high transcript levels of *Fgf8* concomitant with high *Sef* transcript levels. In zebrafish, mouse, and chicken development, *Sef* transcripts mark the MHB and are present in a slightly broader domain than the *Fgf8*-expressing region (Figures 1A–1D, and (Bouchard et al., 2005; Furthauer et al., 2002; Harduf et al., 2005; Lin et al., 2002; Tsang et al., 2002)). Other embryonic tissue regions, including neural tube regions caudal to the MHB, also express *Sef* as part of an FGF8 synexpression group (Harduf et al., 2005). The anti-FZD antibody (OMP-18R5 or Vantictumab, abbreviated 18R5 in this study) binds the conserved extracellular domains of native human FZD1, 2, 5, 7 and 8 proteins (Gurney et al., 2012), transcripts of which are low to absent at the ventral and dorsal midbrain-hindbrain organizer region in mouse E10.5 embryos (Fischer et al., 2007).

In hESC-H9-derived embryoid bodies (EBs), neurally induced (Chambers et al., 2009) and attached to plates coated with poly-ornithine, laminin, and fibronectin (Figure 1K), we identified a SEF^{high}/18R5^{low} population (P4) (Figure 1L). The proportion of this population increased upon treatment with 3 μM CHIR99021 (Figure 1L), a Wnt pathway agonist that induces caudal neural cell fate (Kirkeby et al., 2012a). The caudalization effect of our protocol was confirmed by the down-regulation of forebrain transcripts and up-regulation of hindbrain transcripts (Figure S1). The gene expression profile of 2 μM and 3 μM CHIR99021 treated cultures differed as relatively higher levels of forebrain/midbrain transcripts *WNT1 and OTX2* were present in the former, whereas relatively higher levels of midbrain/hindbrain transcripts *PAX2*, *PAX5*, *FGF17*, and hindbrain transcripts *FGF8* and *GBX2* in the latter (Figure S1). Except for *HOXA2*, similar or higher *HOX* gene transcript levels were present in cultures treated with 3 μM than in cultures treated with 2 μM CHIR99021 (Figure S1). These results indicate the presence of various cell types with a shift from overall more rostral to more caudal cell identities around a MHB profile under these two CHIR99021 concentrations.

Figure 1. Expression patterns of genes at the MHB in embryonic chicken brain sections, and in hESC-H9-derived neurally induced and 3 μM CHIR99021 treated attached EBs, and increase of a SEF^{high}/18R5^{low} population upon Wnt pathway activation.

(A) Schematic overview displaying expression patterns of seven studied genes in sagittal embryonic chicken brain section of developmental stage HH15 as detected by RNA *in situ* hybridization (B–G) and immunostainings (H–J). Regions corresponding to the boxed area in (B) are shown in (C–J). Note the overlap of the *Sef* expression domain with *Fgf8*, *Gbx2*, *En1*, and *Pax2* expression patterns. Scale bars, 100 μm.

(K) Cell culture scheme. hPSCs are kept in culture as floating EBs for 4 days and, upon attachment onto poly-ornithine-, laminin-, and fibronectin-coated plates, cultured for 3 additional days. At day 7, cells are used for further experiments.

(L) (Top) Representative flow cytometry scatterplots of hESC-H9 cells treated with 0, 1, 2, or 3 μM CHIR99021 and labeled with the SEF and 18R5 antibodies. Quadrants were set according to an isotype control. Populations P4–P7 with high and low levels of bound anti-SEF and 18R5 antibodies were gated within the respective quadrants as indicated. (Bottom) Percentages of hESC-H9-derived cells present in the SEF^{high}/18R5^{low} (P4), SEF^{high}/18R5^{high} (P5), SEF^{low}/18R5^{low} (P6), and SEF^{low}/18R5^{high} (P7) populations relative to the total population. Data from three independent experiments (independent biological replicates) are shown. Horizontal bars display the mean values, vertical bars represent standard errors.

(M–X) Distribution of SEF, OTX2, GBX2, PAX2, and FGF8 proteins in neurally induced, 3 μM CHIR99021 caudalized, and attached hESC-H9 EBs as detected by immunostainings, with overlay images in (P, T, and X). SEF expression largely overlaps with GBX2 and PAX2 expression (M, O, Q, and S) and is largely distinct from OTX2 expressing areas (P, T, and X) with only a few OTX2-expressing cells present in the region demarcated by GBX2-, SEF-, and PAX2-expression (P, T, and X). FGF8 expression is present within the GBX2-positive areas (U, W, and X). These representative images are derived from three independent experiments with three technical replicates each. Scale bars, 50 μm.

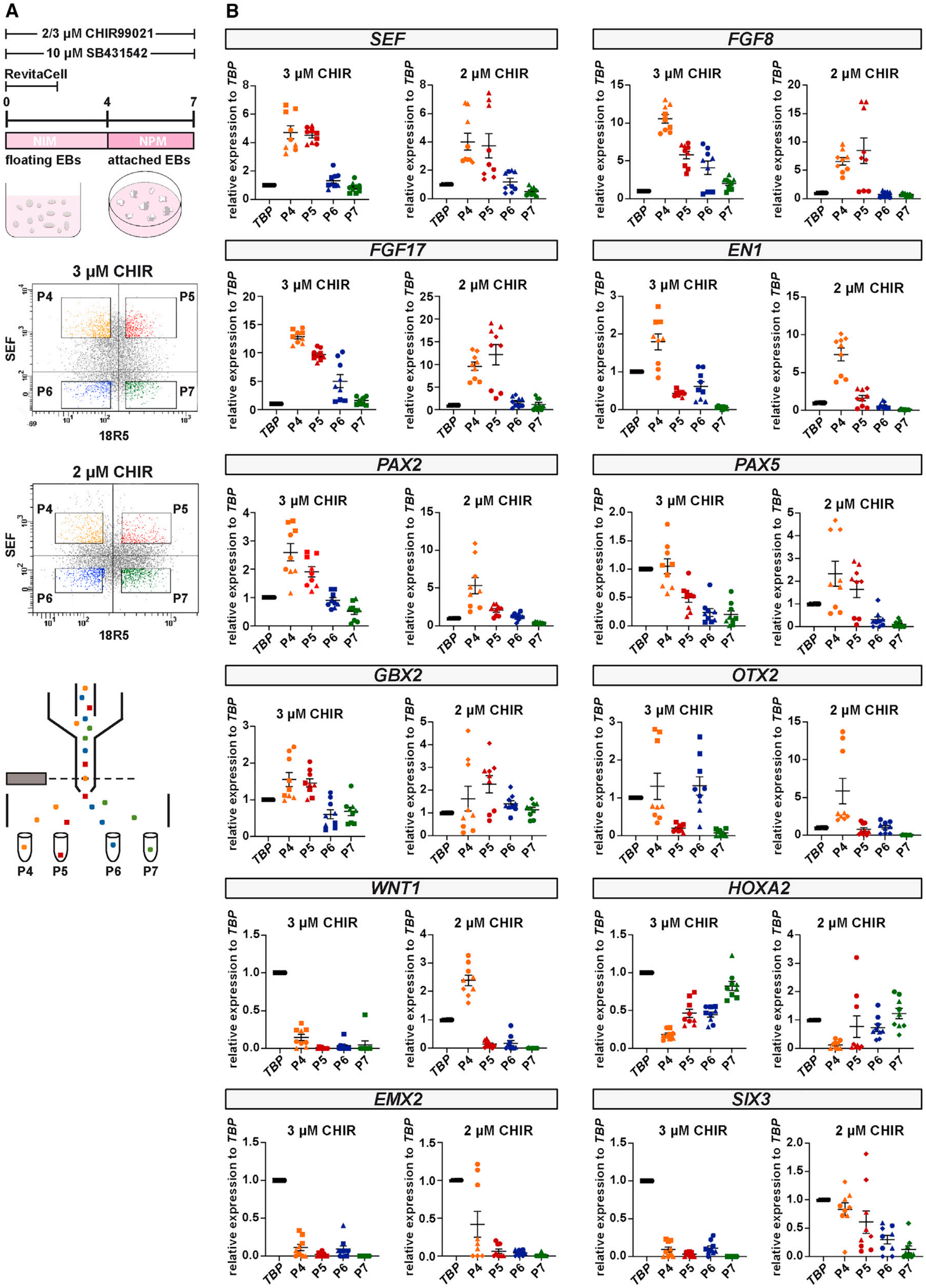

(legend on next page)

Discrete FGF8-producing areas in attached EBs

In neurally induced, 2 μM and 3 μM CHIR99021-treated attached EBs, we identified cell clusters with spatially defined gene expression patterns that resemble the arrangement at the MHB of the developing mouse and chicken brain (3 μM CHIR99021 hESC-H9) (Figures 1M–1X, 3 μM CHIR99021 hiPSC-B4; Figures S2A–S2L, 2 μM CHIR99021 hESC-H9; Figures S2M–S2X, 2 μM CHIR99021 hiPSC-B4; Figures S2A′–S2L′). In 3 μM CHIR99021-treated cultures, we observed largely separate, but directly abutting OTX2-positive and GBX2-positive areas (Figures 1N, 1O, 1V–1X, S2B–S2D, and S2J–S2L), with the latter producing SEF and FGF8 proteins (Figures 1M–1P, 1U–1X, S2A–S2D, and S2I–S2L) and SEF-positive areas also expressing *PAX2* (Figures 1Q–1T, and S2E–S2H). In 2 μM CHIR99021-treated cultures, OTX2- and GBX2-positive areas and OTX2- and SEF-positive areas were predominantly overlapping (Figures S2M–P, S2Q–R, S2A′–D′, S2E′, and S2F′). In such areas, FGF8 and PAX2 immunosignals were present in GBX2-positive or OTX2-negative, but also in OTX2-positive, parts (Figures S2R–S2T, S2U–S2X, S2F′–S2H′, and S2I′–S2L′). In 3 μM CHIR99021-treated cultures, i.e., conditions leading to directly abutting, but largely separate OTX2-positive and GBX2-positive areas, 5.6% ± 1.3% (mean ± SEM) of hESC-H9 EBs and 5.9% ± 0.8% of hiPSC-B4 EBs contained SEF/GBX2-positive areas adjacent to an OTX2-positive territory and 6.7% ± 1.3% and 6.4% ± 1.9% EBs with FGF8/GBX2-positive as well as adjacent OTX2-positive areas were present in hESC-H9 and hiPSC-B4 cultures, respectively. In 2 μM CHIR99021-treated cultures, 30.2% ± 4.2% (mean ± SEM) of hESC-H9 EBs and 18.6% ± 4.3% of hiPSC-B4 EBs contained SEF/GBX2-positive areas adjacent to and overlapping with an OTX2-positive territory and 12.2% ± 1.9% and 10.1% ± 2.6% EBs with FGF8/GBX2-positive, as well as adjacent to and overlapping OTX2-positive, areas were present in hESC-H9 and hiPSC-B4 cultures, respectively (immunostained EBs on three coverslips [technical replicates] from three independent experiments were analyzed per cell line).

SEF^{high}/18R5^{low} gene expression profile

From the total population of neurally induced, 3 μM CHIR99021-treated and attached EB cells, we flow-sorted a SEF^{high}/18R5^{low} population (P4) that contains high transcript levels of genes encoding the midbrain-hindbrain organizer morphogens FGF8 and FGF17. This population is also enriched for other transcripts defining this organizer region in chicken and mouse (such as *SEF*, *EN1*, *PAX2*, *PAX5*, and *GBX2*), which is not the case for the SEF^{high}/18R5^{high} (P5), SEF^{low}/18R5^{low} (P6), and SEF^{low}/18R5^{high} (P7) populations (hESC-H9 [Figure 2] and hiPSC-B4 [Figure S3]). To directly compare the levels of these enriched transcripts in sorted populations to unsorted cells, we performed additional RT-qPCR experiments that also included undifferentiated hPSCs (Figure S4). These data confirmed an up-regulation of these genes in the P4 population. Determined fold changes in comparison with unsorted cells should, however, be interpreted with caution, since we applied narrow sorting gates (Figure 2A). Thus, cells outside these gates are also expected to possess similar expression profiles and are included in the unsorted population. Since we detected increased transcript levels of genes expressed in the rostral MHB region (*WNT1* and *OTX2*), and lower transcript levels of the caudal MHB expressed genes *GBX2* and *FGF8* in the total population of 2 μM compared with 3 μM CHIR99021-treated hESC-H9 cultures (Figure S1), we also analyzed sorted populations from the former cultures (Figure 2). The flow-sorted SEF^{high}/18R5^{low} population (P4) from 2 μM CHIR99021-treated cultures differed from the one obtained under 3 μM CHIR99021 conditions in that relatively higher *WNT1*, *OTX2*, and *EN1* levels and lower *FGF8* and *FGF17* levels were present (Figure 2B).

OTX2 transcript levels in SEF^{high}/18R5^{low} populations isolated from independent cultures varied and immunostainings showed that, whereas 3 μM CHIR99021-treated cultures contained a few GBX2/OTX2-positive, SEF/OTX2-positive, and PAX2/OTX2-positive cells (hESC-H9 [Figures 1M–1X] and hiPSC-B4 [Figures S2A–S2L]), OTX2 and GBX2 territories more extensively overlapped in 2 μM CHIR-treated cultures (Figures S2M–S2L′). Although

Figure 2. The hESC-H9-derived SEF^{high}/18R5^{low} population is enriched for midbrain-hindbrain organizer gene transcripts

(A) Cell culture protocol and flow cytometry-based sorting scheme. hPSCs were kept as floating EBs for 4 days, transferred to poly-ornithine-, laminin-, and fibronectin-coated plates for attachment, and cultured for 3 additional days. Experiments were performed on attached EBs harvested on day 7 and dissociated into single cell suspensions. Representative sorting gates for the isolation of SEF^{high}/18R5^{low} (P4), SEF^{high}/18R5^{high} (P5), SEF^{low}/18R5^{low} (P6), and SEF^{low}/18R5^{high} (P7) populations are shown. Quadrants were set according to an isotype control.

(B) RT-qPCR analyses of the four hESC-H9-derived flow cytometry-sorted cell populations from 2 to 3 μM CHIR99021-treated cultures. Transcript levels of the *TBP* reference gene are set to 1 after normalization to *RFL13A* expression levels. Data from three independent experiments (independent biological replicates) are displayed as filled triangles, circles, and boxes. For each independent biological replicate, three technical replicates (RT-qPCR experiments) were performed. Horizontal bars display the mean values, vertical bars represent standard errors.

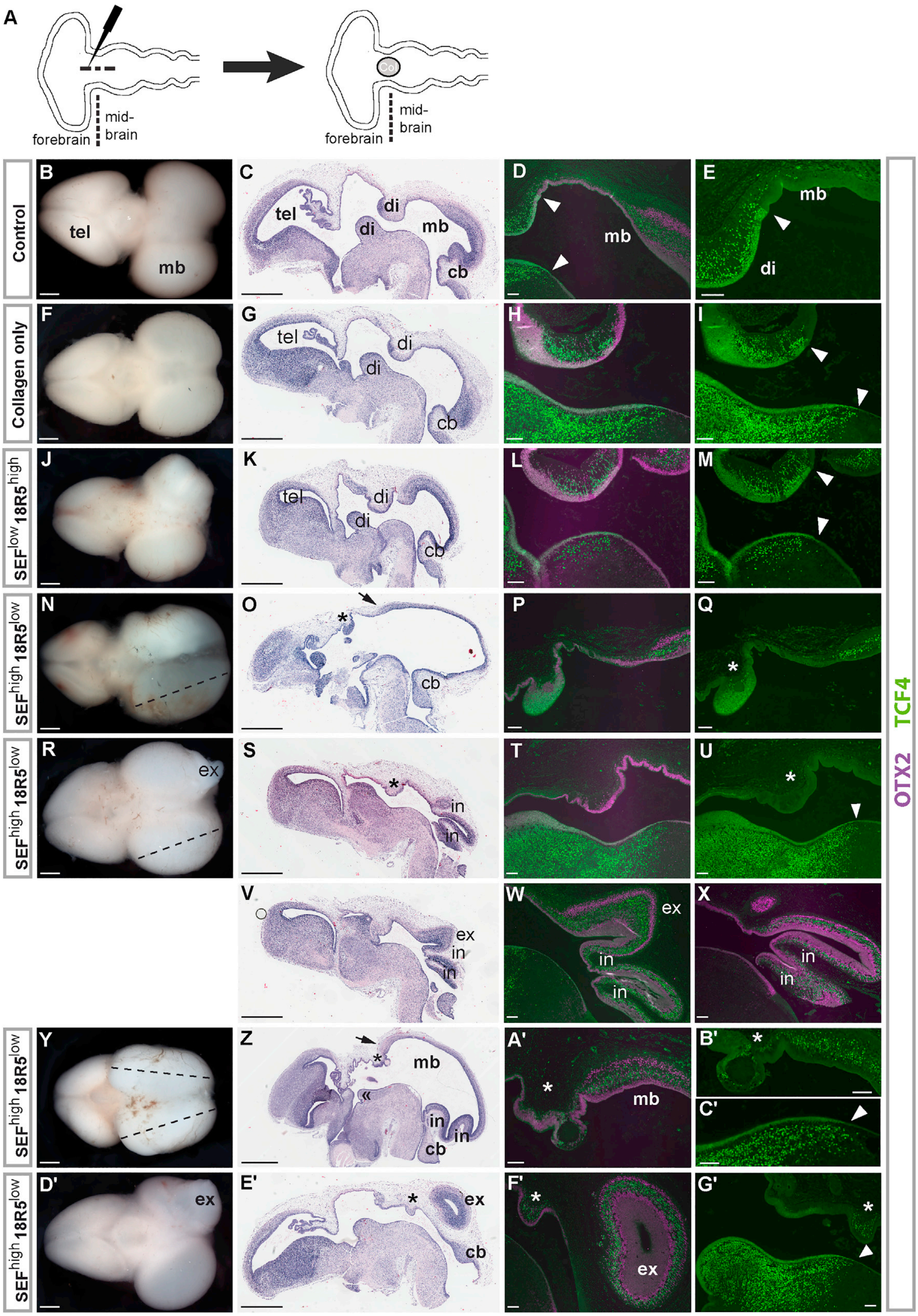

(legend on next page)

we have not performed time course experiments, some of these expression data are reminiscent of MHB development in animals, during which the OTX2 and GBX2 domains initially overlap, but are then distinctly separated at later stages.

We detected very low transcript levels of *HOXA2*, a gene expressed in rhombomere 2 and more caudal rhombomeres, and of the forebrain genes *EMX2* and *SIX3* in the SEF^{high}/18R5^{low} population from 3 μM CHIR99021-treated cultures (hESC-H9 [Figure 2B] and hiPSC-B4 [Figure S3]). Taken together, our results indicate the enrichment of an FGF8-expressing cell population with more rostral (in case of 2 μM CHIR99021) or more caudal (in case of 3 μM CHIR99021) cell identity profiles related to the MHB.

SEF^{high}/18R5^{low} midbrain organizing functionality

Classical transplantation experiments combined with ectopic activation of FGF8 or its related FGF morphogens (by exogenous introduction of beads or expression vectors) led to the discovery of the midbrain-hindbrain organizer and contributed to the understanding of its molecular features (Crossley et al., 1996; Liu et al., 2003; Marin and Puelles, 1994; Martinez et al., 1991, 1995; Nakamura et al., 1986; Sato et al., 2001). Leveraging these experimental approaches, we transplanted sorted hiPSC-B4-derived SEF^{high}/18R5^{low} (P4) and SEF^{low}/18R5^{high} (P7) populations from 3 μM CHIR99021 cultures concentrated in a collagen particle, and collagen particles without cells (three independent sets of transplantations each) into chicken embryos. We placed particles into the caudal diencephalon-anterior midbrain region at the HH developmental stage late 9 to stage 10, stages at which the fate of the neuroepithelium is not yet finally committed, and analyzed embryos 6 days later (see Figure 3A for the transplantation scheme). Upon analysis of the embryos, we found that transplantation of human SEF^{high}/18R5^{low} cells resulted in a rostral elongation of the optic tectum, the dorsal and most prominent chicken midbrain structure in 3 of the 14 surviving embryos (dotted lines in Figures 3N, 3R, and 3Y, and relative length data in Figure 4A). Moreover, localized outward midbrain expansions were present in 2 of the 14 surviving embryos transplanted with SEF^{high}/18R5^{low} cells (labeled 'ex' in Figures 3R, 3V, 3W, and 3D′–F′). There were no similar alterations in the 15 surviving chicken embryos that received collagen only and in the 12 surviving embryos that had received a cell population with low SEF levels (SEF^{low}/18R5^{high}, P7). Four of the 12 surviving SEF^{low}/18R5^{high}-transplanted embryos had malformations (diminished or irregular midbrain), but no elongated dorsal midbrain as observed in SEF^{high}/18R5^{low}-transplanted chicken embryos (compare Figure 3J with Figures 3N, 3R, and 3Y, and see Figure 4A).

In tissue sections, we detected invaginations in two SEF^{high}/18R5^{low} cell-transplanted embryos (labeled "in"; Figures 3S, 3V–3X, and 3Z). Importantly, in four of the SEF^{high}/18R5^{low} cell-transplanted embryos, we found a diminished dorsal diencephalon (indicated by * in Figure 3). In one of those embryos, the ventral diencephalon was also of smaller size (≪ in Figure 3Z). At this stage of chick development, diencephalic tissue can be distinguished from midbrain tissue by TCF4 localization. In the diencephalon, TCF4 is present in the ventricular layer (the innermost layer adjacent to the ventricles) and a supraventricular region; however, in the midbrain, the ventricular layer is TCF4 negative. This sharp difference in TCF4 localization demarcates the diencephalon-midbrain

Figure 3. Transplantation of the hiPSC-B4-derived SEF^{high}/18R5^{low} cell population from 3 μM CHIR99021 treated cultures into the caudal diencephalon-anterior midbrain of late HH9 to HH10 chick embryos induces dorsal midbrain elongation, outgrowth, and invagination

(A) Transplantation scheme.

(B, F, J, N, R, Y, and D′) Brain photos of chick embryos that have developed for another 6 days after late HH9–10 (dorsal view, dotted lines: elongated optic lobes, scale bars, 1 mm).

(C, G, K, O, S, V, Z, and E′) Hematoxylin and eosin-stained sagittal tissue sections. Scale bars, 1 mm.

(D, E, H, I, L, M, P, Q, T, U, A′–C′, F′, and G′) Immunostained sagittal tissue sections encompassing the diencephalon-midbrain boundary region. Scale bars, 100 μm.

(W and X) Immunostained sagittal sections with invaginated and outward expanded midbrain regions. TCF4 immunosignals: green color, OTX2 immunosignals: purple color. Scale bars, 100 μm. cb, cerebellum; di, diencephalon; in, outward midbrain expansion; ex, invaginated dorsal midbrain; mb, midbrain; tel, telencephalon. Arrows point to elongated dorsal midbrain. * and ≪: diminished structures at the putative diencephalon regions. Arrowheads: sharp diencephalon-midbrain boundary marked by the presence/absence of ventricular layer TCF4 at dorsal and ventral regions of a control brain without transplant (D and E), of a brain with a collagen particle transplant (I), of a SEF^{low}/18R5^{high}-transplanted brain (M) and at ventral regions of brains that received SEF^{high}/18R5^{low} cells (U, C′, and G′). Note the reduced TCF4 immunosignals in the ventricular layer, limited to a small structure at the putative dorsal diencephalon region (*) in (Q) and the absence of ventricular layer TCF4 at the dorsal putative diencephalon region (*) in (U, B′, and G′). Multilayered OTX2-TCF4-OTX2-positive normal midbrain tissue (D), elongated midbrain (P, right hand side and A′). (V) More lateral sagittal section than (S) that includes the outward expanded midbrain tissue region. OTX2-TCF4-OTX2-positive multilayer midbrain characteristics of invaginated regions (W, section corresponding with V; X, section corresponding with S) and of outward expanded region (W and F′).

A

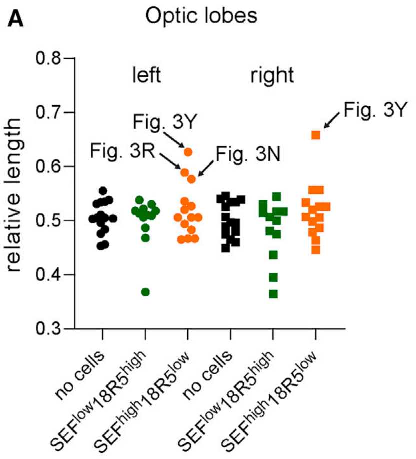

B

	Elongated optic lobes	Reduced diencephalon structures	Loss of sharp diencephalon-midbrain boundary according to TCF4 immunosignals	Invaginated dorsal midbrain	Outward midbrain expansion
**Fig. 3N-Q**	left	dorsal (ventral: not assessed due to tissue damage)	dorsal		
**Fig. 3R-X**	left	dorsal	dorsal	yes	yes
**Fig. 3Y-C'**	right&left	dorsal&ventral	dorsal	yes	
**Fig. 3D'-G'**		dorsal	dorsal		yes

Figure 4. Ratios of rostro-caudal optic lobe length to rostro-caudal whole brain length (relative length) (A) and summary of alterations found in four SEF^{high}18R5^{low} transplant cases (B)

(A and B) Data of left and right optic lobes are shown. Each data point represents one brain. Arrows indicate cases of increased relative optic lobe length. Figure panels showing respective brains are given.

boundary (Merchan et al., 2011), which is found at similar rostro-caudal levels in sagittal sections of dorsal and ventral brain parts (arrowheads in Figure 3D). In addition, a multilayer cytoarchitecture, and the presence of MEIS2 in supraventricular layers, is characteristic of the dorsal midbrain at the investigated stage of chick development (Figure 3D and corresponding part of Figure 5). In the four embryos, ventricular TCF4 immunoreactivity was absent or clearly reduced in the diminished putative dorsal diencephalon tissue areas (Figures 3P, 3Q, 3T, 3U, 3B′, and 3G′). Moreover, MEIS2-positive cells were present in supraventricular positions at elongated dorsal midbrain and diminished putative dorsal diencephalon sites (Figure 5). Supraventricular MEIS2-positive cells were also found in the multilayered invaginations and outward expansions (Figure 5). No contribution from human cells could be found in altered tissue structures based on immunostainings with an anti-human nuclei antibody using tissue sections adjacent to the ones shown in Figure 3. These results indicate that the altered structures represent induced chick tissue.

We did not find any evidence for induced cerebellar structures, either macroscopically or by analyzing tissue sections adjacent to the ones shown in Figure 3 (based on morphology and on PAX6 immunostainings to detect cells of the external granular layer present at this stage of cerebellar development). In previous chick transplantation studies, an actual induction of ectopic cerebellar tissue in the recipient tissue has been achieved in cases where quail organizer tissue was transplanted into the hindbrain (Martinez et al., 1995), but not into a diencephalon-midbrain site (Crossley et al., 1996; Marin and Puelles, 1994; Martinez et al., 1991). High local Fgf8 levels achieved by bead placements (Crossley et al., 1996; Martinez et al., 1999) or electroporation of Fgf8 expression plasmids (Sato et al., 2001) resulted in different outcomes. In cases of sites similar to the one in our transplantation experiments (caudal diencephalon-anterior midbrain), induction of cerebellar-like tissue in addition to midbrain tissue was reported by one study in 50% of cases (Martinez et al., 1999), whereas induction of only mesencephalon, but no cerebellar tissue was found by Sato et al. (2001) and Crossley et al. (1996).

In summary, transplantation of human SEF^{high}/18R5^{low} cells induced the formation of rostrally elongated, outward expanded, and invaginated dorsal midbrain structures, concomitant with smaller diencephalon structures and loss of dorsal diencephalic characteristics. To assess whether the occurrence of four cases with midbrain-organizing effects (summarized in Figure 4B) in only one of the three transplant groups could be a random event, i.e., unrelated to the nature of the transplanted material, we performed a Fisher's exact test with the three groups using the hypergeometric probability distribution. This test yielded a probability of $p = 0.028$ that this result is a random event. The reported anatomical alterations recapitulate results obtained by supplying exogenous FGF8 (FGF8-soaked beads,

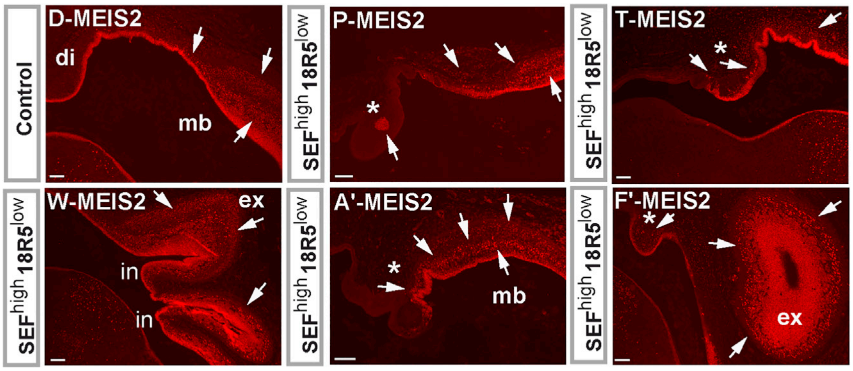

Figure 5. MEIS2 immunosignals (red color) of co-immunostainings corresponding with (D, P, T, W, A′, and F′) OTX2/TCF4 panels

Scale bars, 100 μm. di, diencephalon; ex, outward midbrain expansion; in, invaginated dorsal midbrain; mb, midbrain. *Diminished structures at the putative diencephalon regions. Note the presence of MEIS2 immunosignals (arrows) in supraventricular layers of the control midbrain (D) and in supraventricular positions at elongated dorsal midbrain (P and A′) and diminished putative dorsal diencephalon sites (*). Supraventricular MEIS2-positive cells are also present in the multilayered invaginations and outward expansions (W and F′).

electroporated vectors expressing *Fgf8a*, *Fgf17b*, *Fgf18*, or low levels of *Fgf8b*) into the caudal forebrain-midbrain region of chick embryos; i.e., an expansion of the midbrain optic tectum into the diencephalon territory, interpreted as a diencephalon transformation (see Figure 2D in Crossley et al. [1996]; Figures 2A and 6E in Sato et al. [2001], and Figures 4E–4G in Liu et al. [2003]). The outward midbrain expansions and invaginations are similar to midbrain foliations emerging upon FGF8 bead placement into the diencephalon or midbrain of HH10–12 chicken embryos (see Figure 6 in Shamim et al., 1999).

DISCUSSION

Previous studies show that addition of caudalizing factors to hPSC cultures can lead to the up-regulation of MHB genes (Lee et al., 2018; Muguruma et al., 2015; Wang et al., 2015). However, in these studies, no MHB-like structures were identified, only separate cell aggregates consisting of GBX2-positive cells (indicative of hindbrain identity) without an adjacent OTX2-positive territory (indicative of midbrain identity) (Muguruma et al., 2015; Nayler et al., 2021). The application of a caudalizing CHIR99021 gradient to a tissue layer of neurally induced hESC-H9 cells in a microfluidic system established an OTX2-positive and adjacent OTX2-negative region (Rifes et al., 2020). In this system, peak transcript levels of MHB genes such as *EN1*, *WNT1*, *FGF8*, and *FGF17* were detected in samples dissected around the transition of the OTX2-positive and -negative regions. However, the generated OTX2 boundary was not as clearly defined as it is in animal models, leading the authors to conclude that additional factors are likely needed to refine the boundary. In neurospheres derived from CHIR99021-caudalized and simultaneously ventralized hESC-H9, single cells with an mRNA transcript signature that includes some genes expressed at the MHB (*FGF8*, *FGF17*, *NKX2-8*, and *PAX8*) were identified; however, these were separate from *GBX2*-, *OTX2*-, and *EN1*-expressing cell clusters, and no OTX2-GBX2 boundary tissue was shown (Xu et al., 2022). The success of our method in generating discrete, directly abutting midbrain (OTX2-positive) and hindbrain (GBX2-positive) territories with the presence of additional MHB proteins, could result from several steps: (i) hPSCs were cultured on mouse embryonic fibroblast feeder cells, which secrete a large number of additional factors; (ii) neural induction and caudalization of hPSCs were started simultaneously; (iii) hPSCs exposed to neural induction and caudalization factors had been plated as colony fragments including large aggregates and not as separated, single cells (day 0 in Figure 1K), thereby preserving cell-cell contacts that had formed for a longer period of time, and (iv) the colonies were attached to poly-ornithine, laminin, and fibronectin at day 4 of our protocol and only kept until day 7. The $SEF^{high}/18R5^{low}$ sorting scheme in combination with different levels of CHIR99021 exposure allows the enrichment of a cell population with a gene expression profile resembling more rostral or more caudal cell identities related to the MHB. Since the $SEF^{high}/18R5^{low}$ population is not present as a sharply separated flow cytometry profile, we still consider it a heterogeneous population.

Our transplantation experiments indicate that a human stem cell-derived $SEF^{high}/18R5^{low}$ population produces high enough levels of FGF8 family proteins to display a midbrain-organizing functionality; the low frequency of its *in vivo* midbrain-organizing effect (4 of 14 surviving transplants = 29%) could be due to lower morphogen levels—certainly in comparison with FGF-soaked bead transplantation experiments—and species differences between human and chicken. For example, in xenograft experiments with short-term follow-up, rat and mouse metencephalic tissue was transplanted into the prosencephalon of HH10 chicken embryos and yielded effects in only 21%–43% of the transplantation cases (Martinez et al., 1991). Congenital midbrain-hindbrain malformations in humans

are complex and poorly understood conditions for which disruption of the midbrain-hindbrain organizer has been postulated to underlie several clinical phenotypes (Barkovich et al., 2009; Martinez et al., 2013; Yu et al., 2013).

We establish that surface proteins can be exploited to enrich and transplant *FGF8*-expressing midbrain organizing cells derived from hPSC cultures. As such, this work lays a foundation for studies of normal and impaired human midbrain-hindbrain development. The demonstration of a human *FGF8*-expressing cell population that can be isolated and transplanted may serve as an inspiration to incorporate morphogen-secreting cells for the development of spatially improved hPSC-derived tissue models.

EXPERIMENTAL PROCEDURES

Resource availability

Corresponding author

Ulrike A. Nuber (nuber@bio.tu-darmstadt.de).

Materials availability

The *Sef* encoding plasmid generated in this study is available upon request.

Data and code availability

No standardized datatypes and code were generated.

Ethics statement

Research on surface markers using the hESC-H9 cell line has been approved by the Central Ethics Committee for Stem Cell Research in Germany. E14-E14.5 C57BL/6 mouse embryos for fibroblast isolation: Ethical approval by the local authorities, Regierungspräsidium Darmstadt, Hesse, Germany.

The experiments with chicken embryos of the developmental stages used are not subject to approval under the German Animal Welfare Act. We terminated chick development no later than 6 days after HH10 since there is no sensitivity until day 7 of egg incubation and up to the 10th day of incubation only a limited sensation of the chicken embryo is assumed (Rosenbruch, 1997), and since recognizable alternating cell-dense and cell-sparse layers characteristic for the chick tectum have developed at this stage.

Maintenance of hPSC lines

hiPSC-B4 (Wang and Adjaye, 2011), obtained from James Adjaye (University of Düsseldorf, Germany) and hESC-H9 (WiCell, WA09, RRID:CVCL_9773, obtained from the ES/iPS Facility of the Lund Stem Cell Center, Sweden) were cultured on inactivated C57BL/6 mouse embryonic fibroblasts in 60-mm cell culture dishes (Sarstedt, 83.3901.002) containing 4 mL hPSC medium (DMEM/F12 supplemented with 20% KnockOut serum, 2 mM L-glutamine, 1% non-essential amino acids, 50 μM β-mercaptoethanol and 10 ng/mL basic FGF). Further details are described in the supplemental experimental procedures.

Preparation of mouse embryonic fibroblast feeder cells

Mouse embryonic fibroblasts (MEFs) were isolated from E14–E14.5 C57BL/6 mouse embryos and cultured in MEF medium (DMEM supplemented with 10% FBS, 2 mM L-glutamine, and 1% penicillin-streptomycin) in T75 cell culture flasks (Sarstedt, 83.3911.002) as described previously (Jozefczuk et al., 2012). Further details are given in the supplemental experimental procedures.

Neural differentiation of hPSC lines

hESC-H9 and hiPSC-B4 were cultured for 7 days as described by Kirkeby et al. (2012b) with certain modifications. Floating EB cultures were generated as follows. After aspiration of hPSC medium from hPSC colonies cultured in 60-mm cell culture dishes, 2 mL of 1.5 mg/mL Collagenase IV in DMEM/F12 were added for 30 min at 37°C and colonies were detached using a cell scraper (Corning, 3008) and transferred to a 15-mL tube (Sarstedt, 62.554.502). After detached, fragmented colonies sank to the bottom of the tube, the approximate volume of sedimented colonies was determined. Detached colonies were transferred to single wells (20 μL colony suspension per well) of six-well suspension culture plates (Sarstedt, 83.3920.500) containing 3 mL neural induction medium (1:1 mixture of DMEM/F12 and neurobasal medium supplemented with 1% N2, 2% B27, and 2 mM L-glutamine) per well in the presence of 10 μM SB431542, 1% RevitaCell (days 0–2), 200 μM LDN193189 (only in case of hiPSC-B4 cells), and 0, 1, 2, or 3 μM CHIR99021, and kept on a rocking plate at 80 rpm for 4 days. Medium was changed on day 2. On day 4, floating EBs were transferred to 15 mL tubes (Sarstedt, 62.554.502) and allowed to sink to the bottom of the tube. Sedimented EBs were then plated onto poly-ornithine-, laminin-, and fibronectin-coated six-well plates for cell sorting and total RNA extraction (20 μL EB suspension in 4 mL neural proliferation medium [NPM] per well; Sarstedt, 83.3920), or onto coated glass coverslips in 24-well plates for immunostainings (20 μL EB suspension/6 wells of 24 well plates; Sarstedt, 83.3922; 1 mL NPM per well) and cultured for 3 additional days. NPM consisted of a 1:1 mixture of DMEM/F12 and neurobasal medium supplemented with 0.5% N2, 1% B27, and 2 mM L-glutamine and 10 μM SB431542, 200 μM LDN193189 (the latter in case of hiPSC-B4 cells). CHIR99021 was added to NPM to reach final concentrations of 0, 1, 2, or 3 μM. Coating of wells and coverslips is described in the supplemental experimental procedures.

Fluorescence-activated cell sorting

EB cultures on day 7 of cultivation and cultures of undifferentiated hESC-H9 and hiPSC- B4 were dissociated into single cells by a 6-min Accutase (Table S1) incubation at 37°C followed by mechanical cell separation using a 1,000-μL pipette. The cell suspension was passed through a 40-μm mesh filter (Sarstedt, 83.3945.040) to remove non-dissociated cell clumps. Cell concentrations were measured using a CASY cell counter (Schärfe System, OLS OMNI Life Science) and adjusted to 1 × 10^6 cells/100 μL in fluorescence-activated cell sorting (FACS) buffer (DPBS supplemented with 1% BSA, 25 mM HEPES, 2 mM EDTA, and 1% gentamycin). For information on FACS buffer components, see Table S1. A minimum of 1 × 10^6 cells per vial were incubated with human anti-FZD (1:10,000, OMP-18R5, abbreviated 18R5) and mouse anti-SEF (1:50) antibodies in case of EB cells and with a rat anti-mouse feeder cells antibody (1:50) in case of undifferentiated hPSCs for 30 min at 4°C. Subsequently, cells were washed three times

with 1 mL FACS buffer and incubated for 20 min at 4°C with anti-human-AF488 (1:300) and anti-mouse-PE (1:200) secondary antibodies in case of EB cells. Single stainings with either human 18R5 or mouse anti-SEF primary antibody or a mouse-IgG isotype control antibody (1:200) and the corresponding secondary antibody were prepared for spectral compensation or setting of gates. For further information on antibodies, see Table S2.

After incubation with secondary antibodies, cells were washed three times with 1 mL FACS buffer. Cells were then filtered through a 40-μm cell strainer, 1 μL 25 nM SYTOX Red dead cell stain in BD FACSFlow (Table S1) was added to a 100-μL cell suspension, and cells were immediately analyzed and sorted using a BD FACS Aria III cell sorter and the BD FACS Diva software v8.0 (BD Biosciences). First, dead cells (SYTOX red-positive cells) and non-single cells (based on the width values in forward scatter height against forward scatter width and side scatter height against side scatter width plots) were excluded. Next, SytoxRed-negative (unsorted cells), $SEF^{high}/18R5^{low}$, $SEF^{high}/18R5^{high}$, $SEF^{low}/18R5^{low}$, and $SEF^{low}/18R5^{high}$ populations were sorted in case of a cell density of EB cultures ≥720,000 cells/cm^2. Sorting gates were set to isolate the ~5% highest and lowest populations, respectively. In addition, undifferentiated hPSCs without mouse feeder cells were collected by FACS.

For qPCR analysis, cell populations were sorted directly into lysis buffer containing 4% NP40 alternative (Merck, 492016) and 0.5 U/μL RiboLock RNase-Inhibitor (Thermo Fisher Scientific, EO0381) in nuclease-free water. We sorted 50 cells per μL lysis buffer, incubated on ice for 30 min and centrifuged for 5 min at 13,000×*g* at 4°C. The supernatant was used for cDNA synthesis. For transplantations, cells were sorted into FACS buffer (50,000 cells/500 μL).

Total RNA extraction

Total RNA extraction from hESC H9 and hiPSC B4 cultures neurally induced for 7 days and treated with either 0, 2, or 3 μM CHIR99021 was performed as described in the supplemental experimental procedures.

RT-qPCR analysis

cDNA was synthesized using 12 μL sorted cells in lysis buffer or 1 μg total RNA isolated from 0, 2, or 3 μM CHIR99021-treated cultures using the RevertAid reverse transcriptase (Thermo Fisher Scientific, EP0441) according to the manufacturer's instructions. Briefly, cDNA synthesis was performed in a 20-μL reaction volume containing 0.01 μg/μL Random Hexamers (Thermo Fisher Scientific, SO142), 1 U/μL RiboLock RNase-Inhibitor (Thermo Fisher Scientific, EO0381), and 10 U/μL RevertAid reverse transcriptase at 45°C for 1 h.

RT-qPCR was performed using the PowerUp SYBR Green Master mix (Thermo Fisher Scientific, A25742) according to the manufacturer's instructions. Briefly, qPCR reactions with a total volume of 25 μL containing 400 nM forward and reverse primer and 1 μL cDNA (sorted cells) or 1 μL 1:20 prediluted cDNA (0, 2, or 3 μM CHIR99021-treated cells) were prepared in 96-well PCR plates (Biozym, 710876). qPCR reactions were run on the StepOnePlus Real-Time PCR System (Thermo Fisher Scientific) under the following conditions: 2 min 50°C, 2 min 95°C followed by 40 cycles of 95°C for 15 s and 58°C for 1 min. Primers used for qPCR are listed in the supplemental experimental procedures (Table S3). Data analyses are described in the supplemental experimental procedures.

In situ hybridization of paraffin tissue sections

Brains from chicken embryos stage HH15 (26s) were fixed in 4% PFA at 4°C for 24 h. After three washing steps in PBS for 10 min and a 30-min incubation in 70% EtOH, tissues were stored in 70% EtOH at 4°C until paraffin embedding. We prepared 5 μm sections using a Jung RM 2055 microtome (Leica Biosystems) and were transferred onto Epredia Polysine adhesion glass slides (Epredia, J2800AMNZ). For detailed information on plasmids and RNA probe generation, see the supplemental experimental procedures. Generated Dig-labeled RNA probes were cleaned using the RNeasy Mini Kit. Enzymes and commercial kits used for probe generation are listed in supplemental experimental procedures (Table S4). *In situ* hybridization was performed as described previously (Filatova et al., 2019) with the following modifications: pre-hybridization and hybridization steps were performed at 62°C, and washing was done at 63°C.

Immunostainings of EBs and tissue sections

Immunostainings of PFA-fixed EBs and tissue sections: prior to immunostainings, EB cultures on poly-ornithine-, laminin-, and fibronectin-coated glass coverslips were fixed in 2% PFA for 15 min at room temperature on day 7, followed by two washing steps with PBS. Paraffin tissue sections on glass slides were deparaffinized and an epitope retrieval was performed by boiling glass slides in citrate buffer (10 mM sodium citrate, 0.05% Tween 20, pH 6.0) for 40 min at 90°C and followed by cooling down to room temperature for 45 min.

After a permeabilization step (incubation of coverslips with cultured cells or slides with tissue sections in 0.5% Triton X-100 in PBS for 20 min at room temperature), primary antibodies were applied in 0.01% Triton X-100 in PBS at 4°C overnight. 0.01% Triton X-100/PBS only served as negative control.

Coverslips and slides were washed with PBS three times (10 min each) and incubated with secondary antibodies diluted in 0.01% Triton X-100 and 5% donkey serum (EB cultures only) in PBS at RT for 1 h followed by washing in PBS (10 min, two times). Primary and secondary antibodies and antibody dilutions used are listed in the supplemental experimental procedures (Table S2). Specimens were then incubated in DAPI solution (1 μg/mL, Roche) for 10 min, washed in PBS (5 min), rinsed briefly in water and in 100% ethanol, and air-dried.

Coverslips with attached EBs were mounted on glass slides using Fluorescence Mounting Medium (Agilent, S302380-2). Slides with tissue sections were covered with fluorescence mounting medium and a glass coverslip. Nail polish was used to seal the stained specimens. Pictures were taken using a Zeiss Axiovert 200M microscope or Zeiss Observer D1, and the Zeiss Axiovision software.

Transplantation of sorted cell populations into chicken embryonic brains

Generation of cell-collagen particles: Sorted cell populations in FACS buffer were spun down at 200×*g* for 3 min at 4°C and resuspended in cold DMEM/F12 at a concentration of 25 cells/μL. We transferred 2,500 cells per well into 96 V-shape plates (Sarstedt, 83.3926.500) pretreated with anti-adherence rinsing solution (StemCell Technologies, 07010) for at least 5 min at room temperature. Plates were centrifuged at 200×*g* for 3 min at 4°C to collect

the cells at the center of the conically shaped well bottom resulting in a cell pellet with a diameter of ~400 μm. Next, V-shape plates were put on ice, 20 μL cold collagen type I solution (3 mg/mL; Table S1) was added to each well, and plates were centrifuged at 300×*g* for 4 min at 4°C and afterward incubated at 37°C for 30 min. Plates were transferred to room temperature and cell-collagen particles were taken up with a cut 200-μL filter tip and transferred to a glass slide. Next, a tungsten needle was used to remove as much collagen surrounding the cell pellet as possible.

Transplantation of cell-collagen and collagen-only particles

Fertilized chicken eggs were incubated at 37.8°C and 55%–65% humidity. Eggs were opened, black ink (Pelikan Scribtol, 221135) in PBS was injected underneath the embryo as contrast agent, and somites were counted to determine the developmental stage (HH). Cell-collagen particles containing either sorted $SEF^{high}/18R5^{low}$ or $SEF^{low}/18R5^{high}$ cells and collagen particles without cells were transplanted into chicken embryos at 9 to 10/11 somites (late HH9–10) as follows. A thin tungsten needle was used to make a rostral to caudal cut through the roof plate of the neural tube at the level of the future diencephalon/anterior midbrain. Next, the same tungsten needle was used to place a cell-collagen or collagen-only particle into the neural tube in such a way that the particle occupied the caudal diencephalon and anterior midbrain regions. Eggs were sealed with a tesa strip and incubated for another 6 days. Embryos were then isolated and fixed for 48 h in 4% PFA. Brains were isolated and stored in 70% ethanol at 4°C until further use. Pictures of brains were taken using a Nikon SMZ1500 stereoscopic zoom microscope. Measurement of optic lobe length is described in the supplemental experimental procedures.

Quantification and statistical analysis

The selection of eggs for transplantation and the transplantation order was randomized. The analysis of transplanted embryos was performed in a partially blinded manner. Brains damaged during excision were excluded. To assess whether the occurrence of four cases with midbrain-organizing effects in only one of the three transplant groups could be a random event, Fisher's exact test with the three groups using the hypergeometric probability distribution was performed.

SUPPLEMENTAL INFORMATION

Supplemental information can be found online at https://doi.org/10.1016/j.stemcr.2023.10.007.

ACKNOWLEDGMENTS

We are very grateful to Astrid Vogel-Höpker for experimental advice on transplantations and for comments on the manuscript text. Jutta Smidek-Huhn, Meike Stotz-Reimers, and Michaela Becker-Röck are highly acknowledged for technical assistance. We are deeply indebted to James Adjaye for the provision of the B4 hiPSC line and to Susanne Dietrich, Jose Luis Ferran, and Clemens Kiecker for providing plasmids. Thanks to Dorothea Schulte and Isabella Artner for very valuable comments on the text and figures, and Michael Kohler for advice on statistics.

Funding: German Research Foundation (UN 119/3-1) and the Hessian research promotion program LOEWE (research cluster FLOW FOR LIFE).

AUTHOR CONTRIBUTIONS

Conceptualization, U.A.N.; funding acquisition, U.A.N.; experimental design, N.O., U.A.N., and A.F.; performance of experiments, N.O., A.F., and U.A.N.; data analysis and interpretation, N.O., U.A.N., and A.F.; writing - original draft, U.A.N., and N.O.; writing - review and editing, A.F.

DECLARATION OF INTERESTS

The authors declare no competing interests.

Received: January 30, 2023
Revised: October 5, 2023
Accepted: October 9, 2023
Published: November 2, 2023

REFERENCES

Anderson, C., and Stern, C.D. (2016). Organizers in Development. Curr. Top. Dev. Biol. *117*, 435–454.

Barkovich, A.J., Millen, K.J., and Dobyns, W.B. (2009). A developmental and genetic classification for midbrain-hindbrain malformations. Brain *132*, 3199–3230.

Bouchard, M., Grote, D., Craven, S.E., Sun, Q., Steinlein, P., and Busslinger, M. (2005). Identification of Pax2-regulated genes by expression profiling of the mid-hindbrain organizer region. Development *132*, 2633–2643.

Browne, E.N. (1909). The production of new hydranths in Hydra by the insertion of small grafts. J. Exp. Zool. *7*, 1–23.

Chambers, S.M., Fasano, C.A., Papapetrou, E.P., Tomishima, M., Sadelain, M., and Studer, L. (2009). Highly efficient neural conversion of human ES and iPS cells by dual inhibition of SMAD signaling. Nat. Biotechnol. *27*, 275–280.

Crossley, P.H., Martinez, S., and Martin, G.R. (1996). Midbrain development induced by FGF8 in the chick embryo. Nature *380*, 66–68.

Filatova, A., Rey, L.K., Lechler, M.B., Schaper, J., Hempel, M., Posmyk, R., Szczaluba, K., Santen, G.W.E., Wieczorek, D., and Nuber, U.A. (2019). Mutations in SMARCB1 and in other Coffin-Siris syndrome genes lead to various brain midline defects. Nat. Commun. *10*, 2966.

Fischer, T., Guimera, J., Wurst, W., and Prakash, N. (2007). Distinct but redundant expression of the Frizzled Wnt receptor genes at signaling centers of the developing mouse brain. Neuroscience *147*, 693–711.

Fürthauer, M., Lin, W., Ang, S.L., Thisse, B., and Thisse, C. (2002). Sef is a feedback-induced antagonist of Ras/MAPK-mediated FGF signalling. Nat. Cell Biol. *4*, 170–174.

Gibbs, H.C., Chang-Gonzalez, A., Hwang, W., Yeh, A.T., and Lekven, A.C. (2017). Midbrain-Hindbrain Boundary Morphogenesis: At the Intersection of Wnt and Fgf Signaling. Front. Neuroanat. *11*, 64.

Gurney, A., Axelrod, F., Bond, C.J., Cain, J., Chartier, C., Donigan, L., Fischer, M., Chaudhari, A., Ji, M., Kapoun, A.M., et al. (2012). Wnt pathway inhibition via the targeting of Frizzled receptors results in decreased growth and tumorigenicity of human tumors. Proc. Natl. Acad. Sci. USA *109*, 11717–11722.

Harada, H., Sato, T., and Nakamura, H. (2016). Fgf8 signaling for development of the midbrain and hindbrain. Dev. Growth Differ. *58*, 437–445.

Harduf, H., Halperin, E., Reshef, R., and Ron, D. (2005). Sef is synexpressed with FGFs during chick embryogenesis and its expression is differentially regulated by FGFs in the developing limb. Dev. Dyn. *233*, 301–312.

Jozefczuk, J., Drews, K., and Adjaye, J. (2012). Preparation of mouse embryonic fibroblast cells suitable for culturing human embryonic and induced pluripotent stem cells. J. Vis. Exp.3854

Kirkeby, A., Grealish, S., Wolf, D.A., Nelander, J., Wood, J., Lundblad, M., Lindvall, O., and Parmar, M. (2012a). Generation of regionally specified neural progenitors and functional neurons from human embryonic stem cells under defined conditions. Cell Rep. *1*, 703–714.

Kirkeby, A., Nelander, J., and Parmar, M. (2012b). Generating regionalized neuronal cells from pluripotency, a step-by-step protocol. Front. Cell. Neurosci. *6*, 64.

Lee, J., Choi, S.H., Lee, D.R., Kim, D.S., and Kim, D.W. (2018). Generation of Isthmic Organizer-Like Cells from Human Embryonic Stem Cells. Mol. Cells *41*, 110–118.

Lee, S.M., Danielian, P.S., Fritzsch, B., and McMahon, A.P. (1997). Evidence that FGF8 signalling from the midbrain-hindbrain junction regulates growth and polarity in the developing midbrain. Development *124*, 959–969.

Leto, K., Arancillo, M., Becker, E.B.E., Buffo, A., Chiang, C., Ding, B., Dobyns, W.B., Dusart, I., Haldipur, P., Hatten, M.E., et al. (2016). Consensus Paper: Cerebellar Development. Cerebellum *15*, 789–828.

Lin, W., Fürthauer, M., Thisse, B., Thisse, C., Jing, N., and Ang, S.L. (2002). Cloning of the mouse Sef gene and comparative analysis of its expression with Fgf8 and Spry2 during embryogenesis. Mech. Dev. *113*, 163–168.

Liu, A., Li, J.Y.H., Bromleigh, C., Lao, Z., Niswander, L.A., and Joyner, A.L. (2003). FGF17b and FGF18 have different midbrain regulatory properties from FGF8b or activated FGF receptors. Development *130*, 6175–6185.

Liu, A., Losos, K., and Joyner, A.L. (1999). FGF8 can activate Gbx2 and transform regions of the rostral mouse brain into a hindbrain fate. Development *126*, 4827–4838.

Marin, F., and Puelles, L. (1994). Patterning of the embryonic avian midbrain after experimental inversions: a polarizing activity from the isthmus. Dev. Biol. *163*, 19–37.

Martinez Arias, A., and Steventon, B. (2018). On the Nature and Function of Organizers. Development *145*.

Martinez, S., Andreu, A., Mecklenburg, N., and Echevarria, D. (2013). Cellular and molecular basis of cerebellar development. Front. Neuroanat. *7*, 18.

Martinez, S., Crossley, P.H., Cobos, I., Rubenstein, J.L., and Martin, G.R. (1999). FGF8 induces formation of an ectopic isthmic organizer and isthmocerebellar development via a repressive effect on Otx2 expression. Development *126*, 1189–1200.

Martínez, S., Marín, F., Nieto, M.A., and Puelles, L. (1995). Induction of ectopic engrailed expression and fate change in avian rhombomeres: intersegmental boundaries as barriers. Mech. Dev. *51*, 289–303.

Martinez, S., Wassef, M., and Alvarado-Mallart, R.M. (1991). Induction of a mesencephalic phenotype in the 2-day-old chick prosencephalon is preceded by the early expression of the homeobox gene en. Neuron *6*, 971–981.

Merchán, P., Bardet, S.M., Puelles, L., and Ferran, J.L. (2011). Comparison of Pretectal Genoarchitectonic Pattern between Quail and Chicken Embryos. Front. Neuroanat. *5*, 23.

Muguruma, K., Nishiyama, A., Kawakami, H., Hashimoto, K., and Sasai, Y. (2015). Self-organization of polarized cerebellar tissue in 3D culture of human pluripotent stem cells. Cell Rep. *10*, 537–550.

Nakamura, H., Nakano, K.E., Igawa, H.H., Takagi, S., and Fujisawa, H. (1986). Plasticity and rigidity of differentiation of brain vesicles studied in quail-chick chimeras. Cell Differ. *19*, 187–193.

Nayler, S., Agarwal, D., Curion, F., Bowden, R., and Becker, E.B.E. (2021). High-resolution transcriptional landscape of xeno-free human induced pluripotent stem cell-derived cerebellar organoids. Sci. Rep. *11*, 12959.

Partanen, J. (2007). FGF signalling pathways in development of the midbrain and anterior hindbrain. J. Neurochem. *101*, 1185–1193.

Raible, F., and Brand, M. (2004). Divide et Impera–the midbrain-hindbrain boundary and its organizer. Trends Neurosci. *27*, 727–734.

Rhinn, M., and Brand, M. (2001). The midbrain–hindbrain boundary organizer. Curr. Opin. Neurobiol. *11*, 34–42.

Rifes, P., Isaksson, M., Rathore, G.S., Aldrin-Kirk, P., Møller, O.K., Barzaghi, G., Lee, J., Egerod, K.L., Rausch, D.M., Parmar, M., et al. (2020). Modeling neural tube development by differentiation of human embryonic stem cells in a microfluidic WNT gradient. Nat. Biotechnol. *38*, 1265–1273.

Ron, D., Fuchs, Y., and Chorev, D.S. (2008). Know thy Sef: a novel class of feedback antagonists of receptor tyrosine kinase signaling. Int. J. Biochem. Cell Biol. *40*, 2040–2052.

Rosenbruch, M. (1997). [The sensitivity of chicken embryos in incubated eggs]. ALTEX *14*, 111–113.

Sato, T., Araki, I., and Nakamura, H. (2001). Inductive signal and tissue responsiveness defining the tectum and the cerebellum. Development *128*, 2461–2469.

Shamim, H., Mahmood, R., Logan, C., Doherty, P., Lumsden, A., and Mason, I. (1999). Sequential roles for Fgf4, En1 and Fgf8 in specification and regionalisation of the midbrain. Development *126*, 945–959.

Spemann, H., and Mangold, H. (1924). Uber Induktion yon Embryonalanlagen durch Implantation artfremder 0rganisatoren. Archiv f. mikr. Anat. u. Entwicklungsmechanik *100*, 599–638.

Sunmonu, N.A., Li, K., Guo, Q., and Li, J.Y.H. (2011). Gbx2 and Fgf8 are sequentially required for formation of the midbrain-hindbrain compartment boundary. Development *138*, 725–734.

Tsang, M., Friesel, R., Kudoh, T., and Dawid, I.B. (2002). Identification of Sef, a novel modulator of FGF signalling. Nat. Cell Biol. *4*, 165–169.

Wang, S., Wang, B., Pan, N., Fu, L., Wang, C., Song, G., An, J., Liu, Z., Zhu, W., Guan, Y., et al. (2015). Differentiation of human induced pluripotent stem cells to mature functional Purkinje neurons. Sci. Rep. *5*, 9232.

Wang, Y., and Adjaye, J. (2011). A cyclic AMP analog, 8-Br-cAMP, enhances the induction of pluripotency in human fibroblast cells. Stem Cell Rev. Rep. *7*, 331–341.

Wurst, W., and Bally-Cuif, L. (2001). Neural plate patterning: upstream and downstream of the isthmic organizer. Nat. Rev. Neurosci. *2*, 99–108.

Xu, P., He, H., Gao, Q., Zhou, Y., Wu, Z., Zhang, X., Sun, L., Hu, G., Guan, Q., You, Z., et al. (2022). Human midbrain dopaminergic neuronal differentiation markers predict cell therapy outcomes in a Parkinson's disease model. J. Clin. Invest. *132*, e156768.

Yu, T., Meiners, L.C., Danielsen, K., Wong, M.T., Bowler, T., Reinberg, D., Scambler, P.J., van Ravenswaaij-Arts, C.M., and Basson, M.A. (2013). Deregulated FGF and homeotic gene expression underlies cerebellar vermis hypoplasia in CHARGE syndrome. Elife *2*, e01305.

Zervas, M., Blaess, S., and Joyner, A.L. (2005). Classical embryological studies and modern genetic analysis of midbrain and cerebellum development. Curr. Top. Dev. Biol. *69*, 101–138.

Stem Cell Reports, Volume *18*

Supplemental Information

Enrichment of FGF8-expressing cells from neurally induced human pluripotent stem cell cultures

Nils Offen, Alina Filatova, and Ulrike A. Nuber

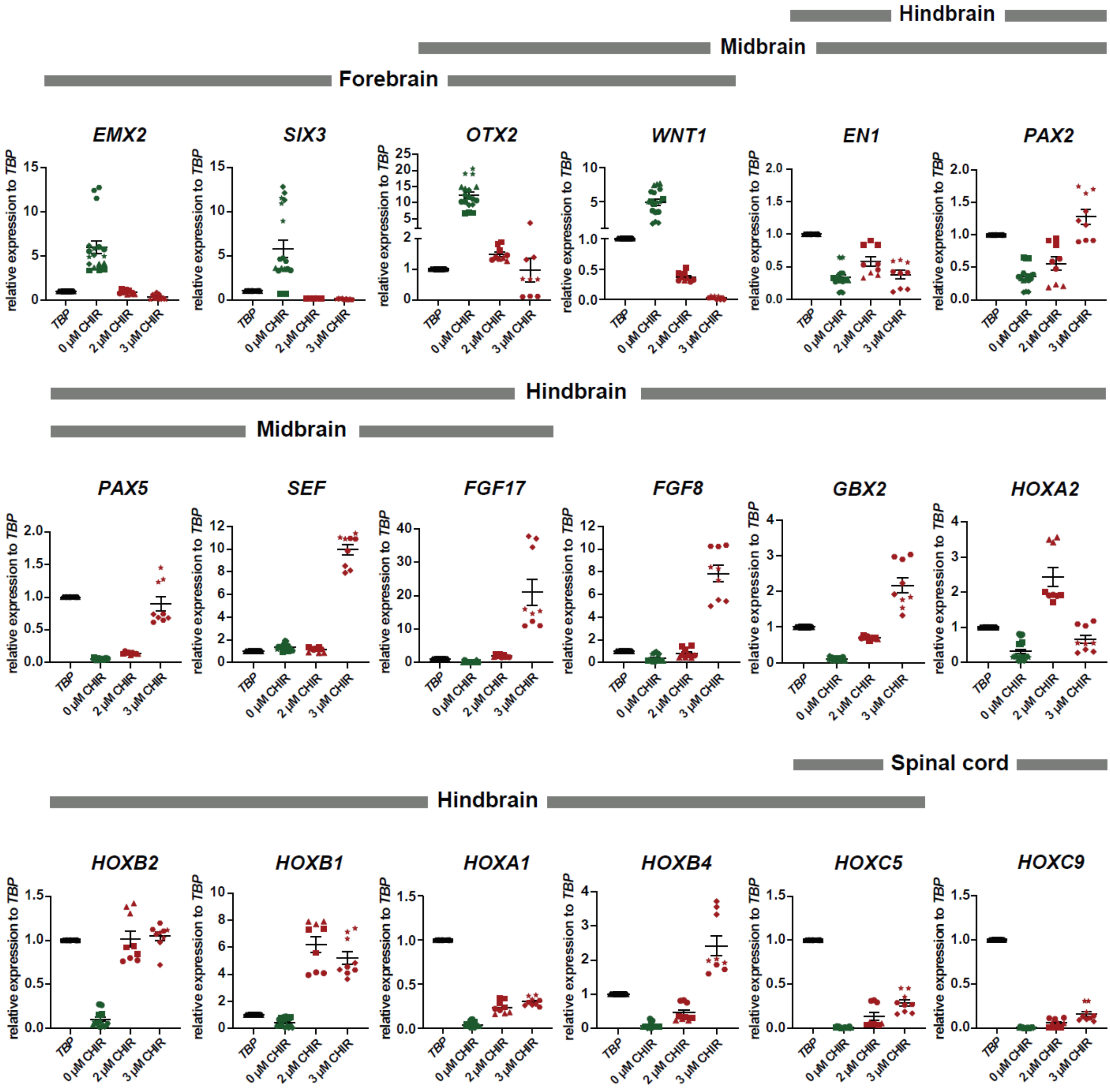

Figure S1. Transcript levels of genes along the anterior-posterior neural tissue axis in neurally induced and attached hESC-H9 EBs treated with 2 or 3 µM CHIR99021 in comparison to untreated samples (0 µM CHIR99021).

Quantitative RT-PCR data from three independent experiments (three independent experiments with 0 and 2 µM CHIR99021 are displayed as triangles, circles, and boxes; three independent experiments with 0 and 3 µM CHIR99021 are displayed as diamonds, stars, and hexagons). For each independent biological replicate, three technical replicates (RT-qPCR experiments) were performed. Horizontal bars display the mean values, vertical bars represent standard errors. Transcript levels of the *TBP* reference gene are set to 1. Note that these genes are not exclusively expressed in indicated brain regions.

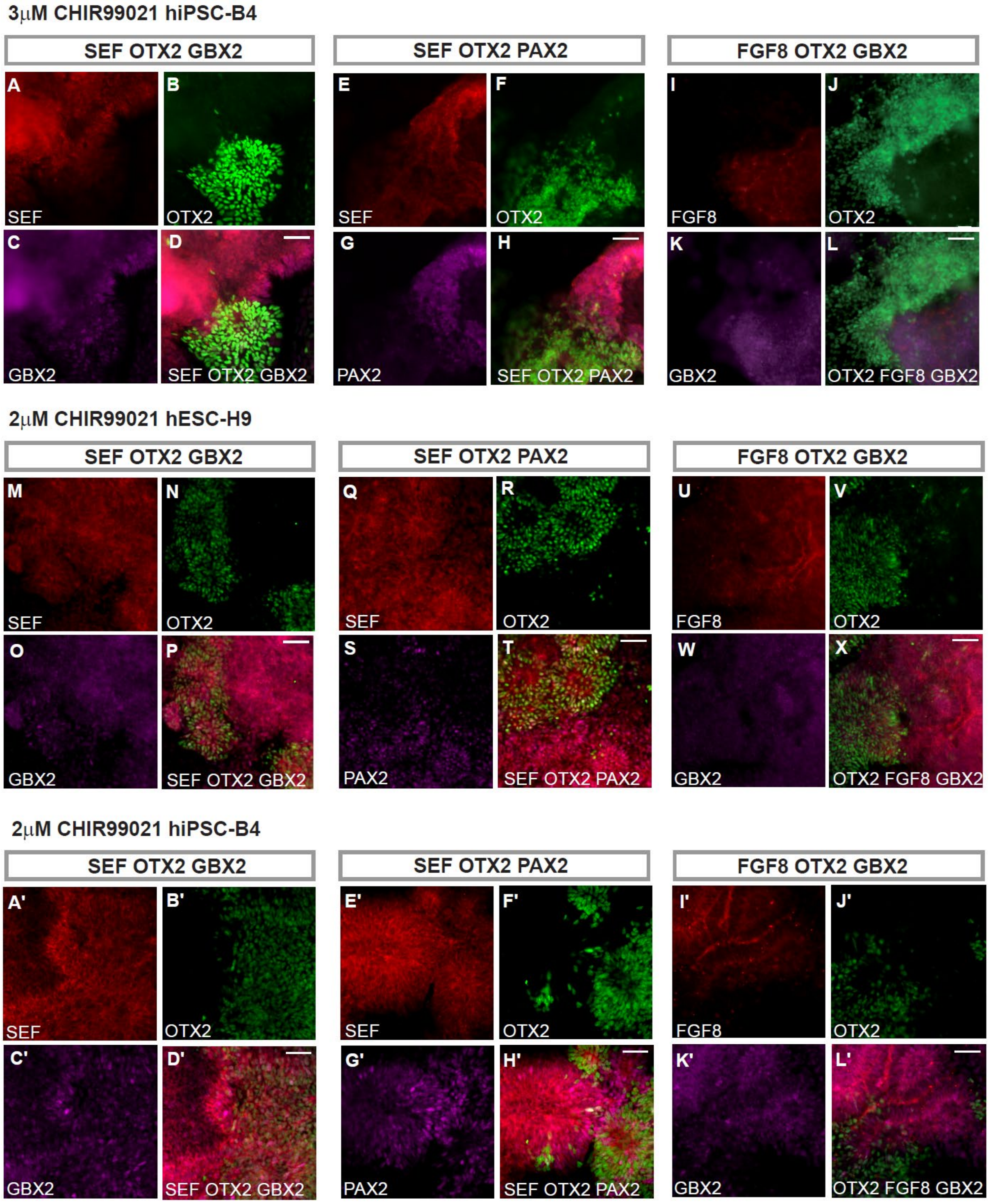

Figure S2. **Distribution of SEF, OTX2, GBX2, PAX2, and FGF8 proteins in neurally induced, caudalized attached EBs studied by co-immunofluorescence stainings**.

Overlay images are shown in the lower right corner of each panel.

(A-L) 3 µM CHIR99021 treated hiPSC-B4, (M-X), 2 µM CHIR99021 treated hESC-H9, (A'-L') 2 µM CHIR99021 treated hiPSC-B4.

In 3 µM CHIR99021 treated hiPSC-B4 cultures, SEF-positive regions are largely overlapping with GBX2- and PAX2-positive areas (A, C, E, G) and are largely distinct from OTX2-positive

areas (D, H). Few OTX2-positive cells are found in the GBX2-, SEF-, and PAX2-positive regions (D, H, L). FGF8-positive regions are present within the GBX2-positive areas (I, K).

In 2 µM CHIR99021 treated hESC-H9 and hiPSC-B4 cultures, SEF-positive regions overlap with GBX2-positive (M, O, A’, C’) and PAX2-positive areas (Q, S, E’, G’). A more extensive overlap of OTX2-positive regions with GBX2-positive/SEF-positive regions is present (M-P, A’-D’). FGF8 immunosignals are detected in GBX2-positive, but also in OTX2-positive areas (U-W, I’-K’). These representative images are derived from three independent experiments per cell line and per CHIR99021 treatment condition with three technical replicates each. Scale bars: 50 µm.

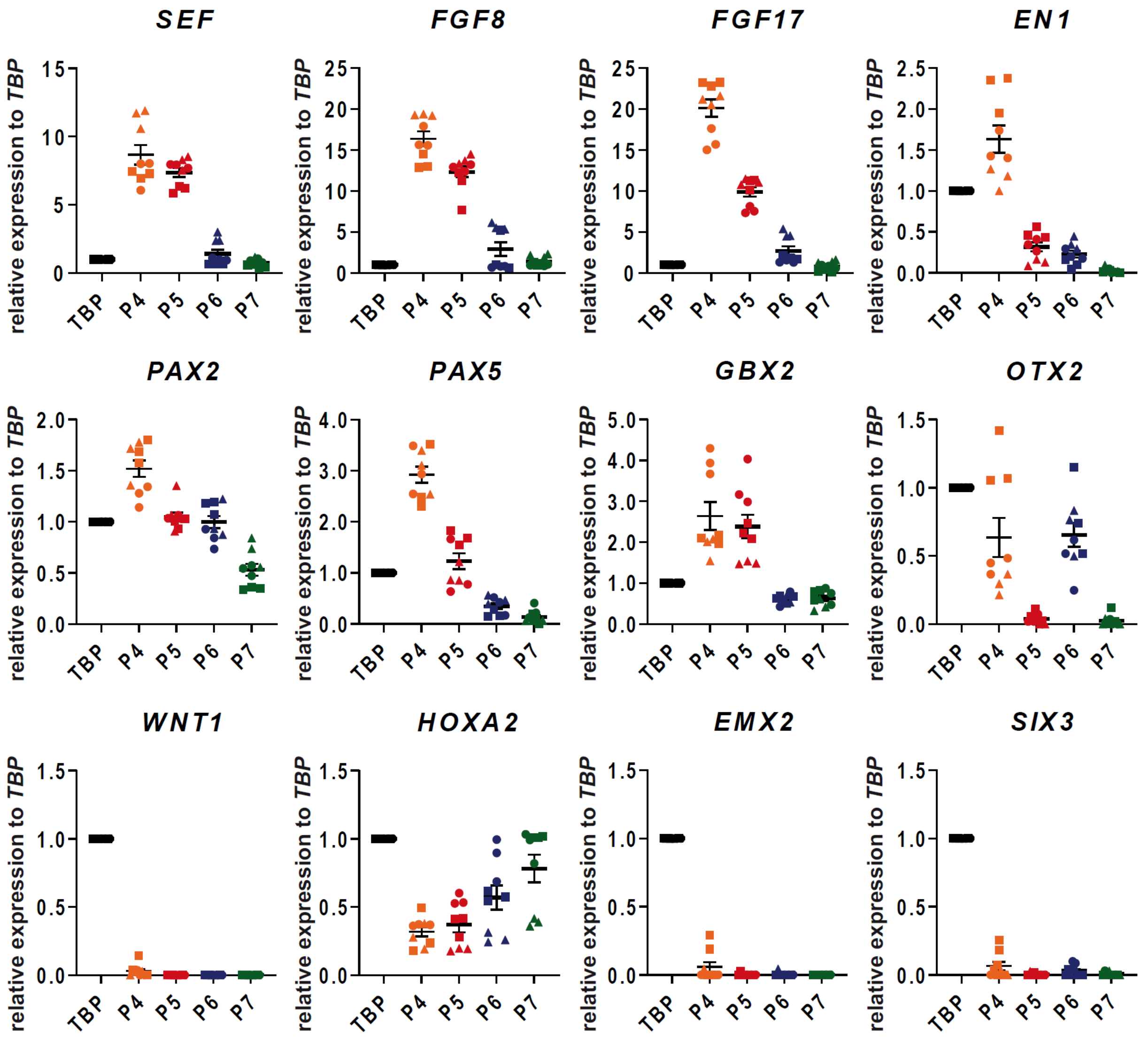

Figure S3. The SEF^{high}/18R5^{low} population isolated from neurally induced, 3 µM CHIR99021 caudalized and attached hiPSC-B4 EBs is enriched for midbrain-hindbrain organizer gene transcripts.

Quantitative RT-PCR analyses of the flow cytometry-sorted cell populations SEF^{high}/18R5^{low} (P4), SEF^{high}/18R5^{high} (P5), SEF^{low}/18R5^{low} (P6), and SEF^{low}/18R5^{high} (P7). Transcript levels of

the reference gene *TBP* are set to 1. Data from three independent experiments (independent biological replicates) are displayed as filled triangles, circles and boxes. For each independent biological replicate, three technical replicates (RT-qPCR experiments) were performed. Horizontal bars display the mean values, vertical bars represent standard errors.

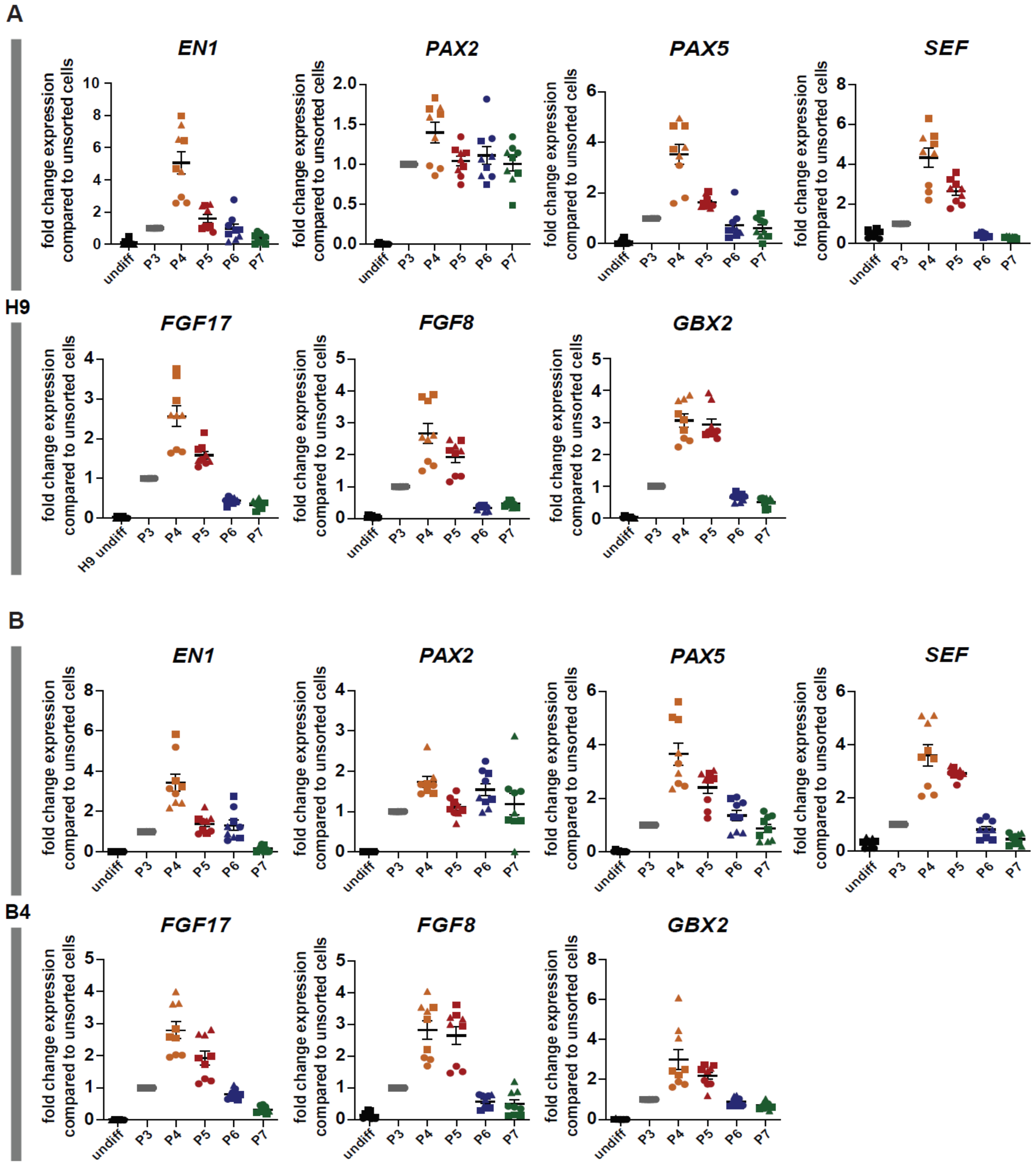

Figure S4. Transcript levels of genes in populations P4, P5, P6, and P7 from 3 µM CHIR99021 treated cultures displayed as fold changes in comparison to the total unsorted population P3 from these cultures.

Quantitative RT-PCR analyses of the flow cytometry-sorted cell populations SEF^{high}/18R5^{low} (P4), SEF^{high}/18R5^{high} (P5), SEF^{low}/18R5^{low} (P6), and SEF^{low}/18R5^{high} (P7), unsorted cell

population (P3), and undifferentiated hPSCs. Data from three independent experiments (independent biological replicates) of hESC-H9 (A) and hiPSC-B4 (B) are displayed as filled triangles, circles, and boxes. For each independent biological replicate, three technical replicates (RT-qPCR experiments) were performed. Horizontal bars display the mean values, vertical bars represent standard errors.

Supplemental experimental procedures

Maintenance of human pluripotent stem cell (hPSC) lines

Lines were weekly passaged and tested for mycoplasma contamination. For passaging, colonies were treated for 5 min with 1.5 mg/ml Collagenase IV in DMEM/F12 at room temperature and detached using a cell scraper. For detailed information on media and supplements, see Table S1.

Preparation of mouse embryonic fibroblast (MEF) feeder cells

MEFs at passage 3 were treated with 10 µg/ml Mitomycin C for 2 h, washed twice with DMEM medium and detached for 5 min with Trypsin-EDTA at 37°C. 4x 10^6 cells per vial were frozen in a 1:1 mixture of MEF medium and FBS supplemented with 10% DMSO. For feeder layers, cells were thawed and replated onto 60 mm cell culture dishes coated with 0.2% gelatin in DPBS for 30 min at 37°C in MEF medium at a seeding density of 30,000-35,000 cells/cm^2. For detailed information on media and supplements, see Table S1.

Neural differentiation of hPSC lines - coating of plates and cover slips

Coating of the bottom of 6 well plates or of 12 mm cover slips (Epredia, CB00120RA120MNZ0) in 24 well plates was performed as follows. 15 µg/ml Poly-ornithine in DPBS (1 ml in case of 6-well plates or 0.5 ml in case of 24-well plates) was added to each well and incubated at 37°C for at least 12 h). After the removal of the poly-ornithine solution, each well was washed twice with 1 (6-well plates) or 0.5 ml (24-well plates) DPBS, respectively. DPBS was removed and DPBS supplemented with 50 µg/ml laminin and 50 µg/ml fibronectin was added to each well (1 ml for 6-well and 0.5 ml for 24-well plates). Plates were incubated at 37°C for 24-48 h, washed twice with either 1 ml (6-well plates) or 0.5 ml (24-well plates) DPBS, and used within a week. For detailed information on media and supplements, see Table S1.

Total RNA extraction

Cells were dissociated into single cells by a 6 min Accutase (Table S1) incubation at 37°C. Cells were rinsed once with DPBS and afterwards either frozen in liquid nitrogen and stored at -80 °C or processed immediately. Total RNA was extracted using the Quick-RNA™ MiniPrep kit (Table S4) according to the manufacturer’s instructions.

RT-qPCR analysis

RT-qPCR data were analyzed using the StepOne software v2.3 (Thermo Fisher Scientific) and Microsoft Excel (Microsoft). RT-qPCR data shown in Figures 2, S1 and S3 were normalized to the reference gene *RPL13A* and presented as $2^{-\Delta CT}{}_{Marker}$ relative to $2^{-\Delta CT}{}_{TBP}$ ± SEM using GraphPad Prism9 (GraphPad Software Inc.). RT-qPCR data shown in Figure S4 were analyzed using the ΔΔCT method. Samples were normalized to the reference gene *RPL13A* and the population P3 (unsorted cells) served as reference population. Data was presented as mean fold change ± SEM compared to the P3 population using GraphPad Prism9.

***In situ* hybridization of paraffin tissue sections**

The following plasmids were used to generate RNA *in situ* probes: *En1* (from Susanne Dietrich), *Fgf8* (from Astrid Vogel-Höpker), *Gbx2* (from Jose Luis Ferran), *Wnt1* (from Clemens Kiecker) and *Sef*. To generate the *Sef* encoding plasmid, a 530 bp cDNA fragment corresponding to nucleotides 1016-1545 of Genbank: XM_040646074.1 was PCR-amplified using a forward (5'-TCTAGAATTCTCCCTGTG GAAAGACTTCGC-3') and reverse primer (5'-TTTCTAGAACACCTCTGAATCCTGC ACAC-3') and cloned into pBluescriptII SK+ via *BamHI* and *XbaI* sites. All plasmids were sequenced to confirm respective cDNA inserts.

To generate Dig-labeled RNA probes, a DNA fragment with a corresponding cDNA sequence of interest and the T3, SP6 or T7 promoter sequence, was amplified by PCR. PCR amplification was performed in 50 µl reactions containing 200 nM of forward (5'-CCTCTTCGCTATTACGCCAG-3') and reverse primer (5'-GGCTCGTATGTTGTGTGGAA-3'), 200 µM dNTPs and 0.2 U/µl Q5 High Fidelity DNA polymerase in an GeneAmp 9600 PCR cycler (Applied Biosystems) under the following conditions. 2 min 98°C, followed by 35 cycles 10 sec 98°C, 20 sec 56°C and 1 min 30 sec 72°C and a final elongation step for 2 min at 72°C. PCR products were purified using the DNA Clean & Concentrator-5 kit according to the manufacturer's instructions. 1 µg of DNA was used for *in vitro* transcription with the following RNA polymerases: *En1* and *Sef,* T7; *Fgf8* and *Wnt1*, T3; *Gbx2*, Sp6.

Measurement of optic lobe length

Lateral photos of isolated embryonic chicken brains transplanted with either collagen-SEF^{high}/18R5^{low} cell particles or collagen-only were taken at day 6 post transplantation

using a Nikon SMZ1500 stereoscopic zoom microscope. Fiji (Schindelin et al., 2012) was used to measure the rostro-caudal length of the whole brain and of the optic lobe. Data were presented as ratios of optic lobe length to whole brain length using Graphpad Prism.

Table S1: Media and supplements used for cell culture and FACS.

Medium/Supplement	Distributor	Cat. No.
Accutase	Sigma-Aldrich	A6964
Albumin Fraktion V (BSA)	Carl Roth	CP84.2
B27 supplement without vitamin A (50x)	Thermo Fisher Scientific	12587-010
ß-mercaptoethanol	Thermo Fisher Scientific	31350-010
Collagen, Type I solution from rat tail (3 mg/ml)	Sigma-Aldrich	C3867
Collagenase IV	Thermo Fisher Scientific	17104-019
CHIR99021	Sigma-Aldrich	SML1046
DMEM high Glucose	Sigma-Aldrich	D5671
DMEM/F12	Sigma-Aldrich	51445C
DPBS	Sigma-Aldrich	D8537
DMSO	Sigma-Aldrich	D4540
UltraPure™0.5 M EDTA, pH 8.0	Thermo Fisher Scientific	15575020
BD FACSFlow™ Sheath Fluid	BD Biosciences	342003
FBS	Sigma-Aldrich	F7524
Recombinant human FGF basic (154 a.a.)	Peprotech	100-18B
Fibronectin	Thermo Fisher Scientific	33010-018
Gelatin	Sigma-Aldrich	G1890
Gentamycin	Sigma-Aldrich	G1397
1M HEPES solution	Thermo Fisher Scientific	15630-080
KnockOut Serum Replacement	Thermo Fisher Scientific	10828-010
Laminin	Sigma-Aldrich	L2020
LDN193189 hydrochloride	Sigma-Aldrich	SML0559
L-glutamine	Sigma-Aldrich	G7513
MEM non-essential amino acids	Sigma-Aldrich	M7145
Mitomycin C	Roche	10107409001
N2 supplement (100x)	Thermo Fisher Scientific	17502-048
Neurobasal medium	Thermo Fisher Scientific	21103-049
Penicillin-Streptomycin	Sigma-Aldrich	P0781

Poly-L-ornithine hydrobromide	Sigma-Aldrich	P3655
RevitaCell™ supplement (100x; Rho-associated kinase inhibitor)	Thermo Fisher Scientific	A2644501
StemMACS™ SB431542	Miltenyi Biotec	130-106-275
SYTOX™ Red dead cell stain	Thermo Fisher Scientific	S34859
0.5% Trypsin/ 0.2% EDTA	Sigma-Aldrich	T3924

Table S2: Primary and secondary antibodies.

Antibody	Distributor	Cat. No.	Stock concen-tration	Dilution for immuno-stainings
**Primary antibodies**				
sheep anti-Digoxigenin-AP, RRID:AB_2313640	Roche	11 093 274 910	0.75 U/µl	1:1500
mouse anti-FGF8, RRID:AB_2927654	Creative Biolabs	NEUT-823CQ	0.5 mg/ml	1:500
human anti-FZD (Vantictumab), RRID:AB_2911149	Thermo Fisher Scientific	MA5-42006	1.1 mg/mL	1:10000
rabbit anti-GBX2, RRID:AB_2685900	Sigma-Aldrich	HPA067809	900 µg/ml	1:500
mouse anti-MEIS2, RRID:AB_2618842	DSHB	PCRP-MEIS2-1A11	21 µg/ml	1:10
goat anti-OTX2, RRID:AB_2157172	R&D Systems	AF1979	200 µg/ml	1:500
mouse anti-PAX2, RRID:AB_2722284	DSHB	PCRP-PAX2-1A7	12 µg/ml	1:10
rabbit anti-PAX2, RRID:AB_2734656	Biolegend	901002	1 mg/ml	1:500
rabbit anti-PAX6, RRID:AB_2565003	Biolegend	901301	2 mg/ml	1:500
mouse anti-IL-17RD/SEF, RRID:AB_2125688	R&D Systems	MAB2275	250 µg/ml	1:200
rabbit anti-TCF4/TCF7L2, RRID:AB_2199816	Cell Signaling Technology	2569	-	1:200
Rat anti-mouse feeder cells antibody, RRID:AB_2752027	Miltenyi Biotec	130-120-166	150 µg/ml	only used for FACS
Mouse anti-human nuclei antibody, RRID:AB_94090	EMD Millipore	MAB1281	-	1:200

**Secondary antibodies**				
anti-goat-AF488, RRID:AB_2336933	Dianova	705-545-147	1 mg/ml	1:500
anti-goat-AF647, RRID:AB_2535864	Thermo Fisher Scientific	A-21447	1 mg/ml	1:500
anti-human-AF488, RRID:AB_2340565	Dianova	709-545-098	1 mg/ml	FACS only
anti-mouse-Cy3, RRID:AB_2340813	Dianova	715-165-150	1 mg/ml	1:300-1:500
anti-rabbit-Cy5, RRID:AB_2340607	Dianova	711-175-152	1 mg/ml	1:500-1:1000
anti-goat-Cy3, RRID:AB_2307351	Dianova	705-165-147	1 mg/ml	1:300
anti-mouse DyLight 488, RRID:AB_2687442	Dianova	715-485-150	1.5 mg/ml	1:300
anti-rabbit-AF488, RRID:AB_2313584	Dianova	711-545-152	1 mg/ml	1:300
anti-mouse-RPE, RRID:AB_2338622	Dianova	115-115-207	1 mg/ml	only used for FACS
**Flow cytometry isotype control**				
mouse IgG2B isotype control, RRID:AB_357346	R&D Systems	MAB004	1 mg/ml	only used for FACS

Table S3: RT-qPCR primers.

Gene	Primer	Sequence (5' → 3')
EMX2	EMX2-fw	GGATCCGTCCACCTTCTACC
	EMX2-rev	CGTGTTCCAGCCTTAGAAGC
EN1	EN1- fw	CTGCACACGTTATTCGGATCG
	EN1-rev	CGCTTGTCCTCCTTCTCGTTC
FGF8	FGF8-fw	CAACTCTACAGCCGCACCAG
	FGF8-rev	TGCTTCCAAAGGTGTCCGTC
FGF17	FGF17-fw	GCAGATCCGCGAGTACCAAC
	FGF17-rev	GTCCGTCTCCACTATGAGCTTG
GBX2	GBX2-fw	AGGACGGCAAAGGCTTCCTG
	GBX2-rev	GACTCGTCTTTCCCTTGCC
HOXA1	HOXA1-fw	TCGCCTCAATACATTCACCACT
	HOXA1-rev	TTGACCCAGGTAGCCGTACT
HOXA2	HOXA2-fw	CTTGCCTCAGCCACAAAGAATC
	HOXA2-rev	GCTGTGTGTTGGTGTAAGCAG
HOXB1	HOXB1-fw	CAGAACCTAACACCCCCACG
	HOXB1-rev	AGTTCTGTCAGCTGCCTTGT
HOXB2	HOXB2-fw	AATCCGCCACGTCTCCTTC
	HOXB2-rev	CAGCTGCGTGTTGGTGTAAG
HOXB4	HOXB4-fw	CTACCCCTGGATGCGCAAAG
	HOXB4-rev	GTCAGGTAGCGGTTGTAGTGA
HOXC5	HOXC5-fw	TACCCGTGGATGACCAAACTG
	HOXC5-rev	GAGACACAAGTTGTTGGCGA
HOXC9	HOXC9-fw	CCGGCAGCAAGCACAAAGA
	HOXC9-rev	TACCGACGGTCCCTGGTTAAA

OTX2	OTX2-fw	CGGTACCCAGACATCTTCATG
	OTX2-rev	CGGCACTTAGCTCTTCGATTC
PAX2	PAX2-fw	GACTATGTTCGCCTGGGAGATTC
	PAX2-rev	AAGGCTGCTGAACTTTGGTCCG
PAX5	PAX5-fw	GTAGTCCGCCAGAGGATAGTG
	PAX5-rev	TCCAATTACCCCAGGCTTGATG
RPL13A	RPL13A-fw	GTATGCTGCCCCACAAAACC
	RPL13A-rev	TTCAGACGCACGACCTTGAG
SEF	SEF-fw	CGACGCTCTTCACTGTGATGTG
	SEF-rev	GCTGCAGTGTATGTGGAAGAC
SIX3	SIX3-fw	AGAACAGGCTCCAGCACCAG
	SIX3-rev	CTGGAGGTTACCGAGAGGATG
TBP	TBP-fw	CTTGTGCTCACCCACCAAC
	TBP-rev	CTGCTCTGACTTTAGCACCTG
WNT1	WNT1-fw	CCGATGGTGGGGTATTGTGA
	WNT1-rev	TCCCCGGATTTTGGCGTATC

Table S4: Enzymes and commercial kits used for *in situ* hybridization probe generation and total RNA extraction.

Enzyme	Distributor	Cat. No.
Q5 High Fidelity DNA polymerase	NEB	M0491S
SP6 Polymerase	New England Biolabs (NEB)	M0207S
T3 Polymerase	New England Biolabs (NEB)	M0378S
T7 Polymerase	New England Biolabs (NEB)	M0251S
DNA Clean & Concentrator-5 kit	Zymo Research	D4004
Quick-RNA™ MiniPrep kit	Zymo Research	R1054
RNeasy Mini Kit	Qiagen	74104

Reference

Schindelin, J., Arganda-Carreras, I., Frise, E., Kaynig, V., Longair, M., Pietzsch, T., Preibisch, S., Rueden, C., Saalfeld, S., Schmid, B., *et al.* (2012). Fiji: an open-source platform for biological-image analysis. Nat Methods *9*, 676-682.
